# In vivo binding of PRDM9 reveals interactions with noncanonical genomic sites

**DOI:** 10.1101/gr.217240.116

**Published:** 2017-04

**Authors:** Corinne Grey, Julie A.J. Clément, Jérôme Buard, Benjamin Leblanc, Ivo Gut, Marta Gut, Laurent Duret, Bernard de Massy

**Affiliations:** 1 Institut de Génétique Humaine UMR9002 CNRS-Université de Montpellier, 34396 Montpellier Cedex 05, France;; 2 Biotech Research and Innovation Centre (BRIC), University of Copenhagen, 2200 Copenhagen, Denmark;; 3 CNAG-CRG, Centre for Genomic Regulation (CRG), Barcelona Institute of Science and Technology (BIST), 08028 Barcelona, Spain;; 4 Universitat Pompeu Fabra (UPF), 08003 Barcelona, Spain;; 5 Université de Lyon, Université Claude Bernard, CNRS, Laboratoire de Biométrie et Biologie Evolutive UMR 5558, F-69100, Villeurbanne, France

## Abstract

In mouse and human meiosis, DNA double-strand breaks (DSBs) initiate homologous recombination and occur at specific sites called hotspots. The localization of these sites is determined by the sequence-specific DNA binding domain of the PRDM9 histone methyl transferase. Here, we performed an extensive analysis of PRDM9 binding in mouse spermatocytes. Unexpectedly, we identified a noncanonical recruitment of PRDM9 to sites that lack recombination activity and the PRDM9 binding consensus motif. These sites include gene promoters, where PRDM9 is recruited in a DSB-dependent manner. Another subset reveals DSB-independent interactions between PRDM9 and genomic sites, such as the binding sites for the insulator protein CTCF. We propose that these DSB-independent sites result from interactions between hotspot-bound PRDM9 and genomic sequences located on the chromosome axis.

Recombination between homologous chromosomes is required for proper chromosome segregation at the first meiotic division in the majority of sexually reproducing organisms. This specific recombination pathway is initiated by the formation of DNA double-strand breaks (DSBs) (de Massy 2013) that are generated by the SPO11/TOPOVIBL protein complex (Robert et al. 2016; Vrielynck et al. 2016) and repaired by homologous recombination (Baudat et al. 2013; Hunter 2015). DSB repair leads to reciprocal and nonreciprocal exchanges of genetic material between paternal and maternal chromosomes, called cross-overs and gene conversions, respectively. Meiotic recombination is essential for fertility in most species and is a major source of genome diversity (Chen et al. 2007; Coop and Przeworski 2007).

Meiotic recombination takes place during extensive chromosome reorganization at meiotic prophase. Chromosomes arrange as an array of chromatin loops that are anchored to a protein axis, made of cohesins and other structural proteins. This structure serves as a platform for various members of the recombination machinery and for regulating the recombination activity (Zickler and Kleckner 1999). This loop-axis configuration plays an important role in the regulation of DSB formation via induction (Borde and de Massy 2013) and inhibition (Thacker et al. 2014) of DSB activity.

In *Saccharomyces cerevisiae*, where DSB sites have been analyzed in detail, DSBs preferentially occur in loops and in accessible nucleosome-depleted chromatin regions of ∼200 bp, which are called DSB hotspots and which are mainly located within promoter regions, upstream of transcription start sites. Hotspots are flanked by positioned nucleosomes that are enriched in trimethylation of lysine 4 of histone H3 (H3K4me3) (Pan et al. 2011). This histone modification is deposited by SET1, a subunit of the COMPASS complex recruited by RNA polymerase II. Although not absolutely required, H3K4me3 is important for normal DSB levels and localization (Borde et al. 2009). SPP1, a member of the SET1 complex binds to H3K4me3 and to MER2, an essential member of the meiotic DSB formation machinery (Acquaviva et al. 2013; Sommermeyer et al. 2013), located on the axis of meiotic chromosomes (Panizza et al. 2011). The SPP1-mediated interactions are thought to tether and/or stabilize the hotspot region to the axis where DSBs are predicted to occur (Blat et al. 2002; Panizza et al. 2011).

In mice and humans, meiotic recombination hotspots are determined by the DNA sequence specificity of the PRDM9 zinc finger domain (Baudat et al. 2010; Myers et al. 2010; Parvanov et al. 2010). Different from *S. cerevisiae*, these hotspots are not preferentially located in promoter regions, but they are enriched in H3K4me3 (Buard et al. 2009; Smagulova et al. 2011), presumably through PRDM9 methyltransferase activity (Hayashi et al. 2005). Indeed, PRDM9 can methylate histone H3 at K4, K9, and K36 in vitro (Wu et al. 2013; Eram et al. 2014; Koh-Stenta et al. 2014). Like H3K4me3, H3K36me3 also can be associated with nucleosomes adjacent to hotspots (Buard et al. 2009; Davies et al. 2016; Powers et al. 2016). Hotspot-associated H3K4me3 is detected as early as 9 d post-partum (dpp) in mouse testis when spermatocytes begin to enter leptonema, the first stage of meiotic prophase (Grey et al. 2011). This H3K4me3 enrichment does not depend on *Spo11*, consistent with a role before DSB formation (Buard et al. 2009; Grey et al. 2011; Brick et al. 2012; Borde and de Massy 2013). However, it has been hypothesized that H3K36me3 is involved in DSB repair by homologous recombination in mammalian cells (Aymard et al. 2014; Carvalho et al. 2014; Pfister et al. 2014), thus raising the question of when and how this mark is deposited and whether its deposition depends on DSB formation.

A remarkable feature of PRDM9 is its C2H2 zinc finger domain that enables the protein to recognize specific DNA motifs and to tether initiation of meiotic recombination to specific sites in the genome. This zinc finger domain, which has a minisatellite-like structure, is highly polymorphic in mice and humans (Berg et al. 2010; Buard et al. 2014; Kono et al. 2014) and mutates at a high rate in the human germ line (Jeffreys et al. 2013). *Prdm9* zinc finger alleles differ mostly by the number of zinc fingers or by substitutions of amino acids involved in the interaction with DNA, thus leading to variability in the DNA sequence specificity. In mice and humans, it has been directly demonstrated that meiotic DSBs are specified by PRDM9 by chromatin immunoprecipitation and sequencing (ChIP-seq) of the binding sites of the DMC1 strand invasion protein. DMC1 binds to single-strand DNA generated by DSB end processing (Smagulova et al. 2011). By use of this approach, it was shown, in mice and humans, that PRDM9 variants with different zinc finger arrays specify distinct, essentially not overlapping, sets of meiotic DSB sites throughout the genome (Brick et al. 2012; Pratto et al. 2014; Smagulova et al. 2016). PRDM9, which belongs to the PRDM family of transcription regulators (Fog et al. 2012), has homologs in most mammals with the exception of *Canidae*. However, PRDM9 is absent, is not functional, or is not clearly identifiable in all other vertebrate lineages examined to date. In dogs and birds, where PRDM9 is nonfunctional or absent, meiotic hotspots preferentially localize to functional genomic elements that are enriched in H3K4me3, such as transcription start sites and/or CpG islands, a chromatin environment sharing similarities with *S. cerevisiae* DSB sites (Munoz-Fuentes et al. 2011; Axelsson et al. 2012; Auton et al. 2013; Singhal et al. 2015). Strikingly, when *Prdm9* is inactivated in mice, DSB formation still occurs, but at new locations that mainly correspond to promoter regions, which are also enriched in H3K4me3 (Brick et al. 2012). However, for unknown reasons, the downstream repair pathway is partially defective and meiotic progression is altered. Thus, in the mouse, PRDM9 is indispensable for normal fertility (Hayashi et al. 2005).

Here, we performed PRDM9 ChIP-seq using chromatin from mouse testes to analyze PRDM9 binding sites and to evaluate the relationship between PRDM9 sites and meiotic DSB formation. For this, we used two mouse strains with different *Prdm9* alleles with distinct zinc finger domains. We mapped H3K4me3, H3K36me3, and DMC1 distribution by ChIP-seq in the same strains. We also investigated the functional relationship between DSB formation, PRDM9 binding, and H3K36me3 enrichment by analyzing PRDM9 binding and H3K36me3 enrichment in *Spo11*-deficient mice.

## Results

### Identification of two classes of PRDM9 binding sites

**Figure 1.**
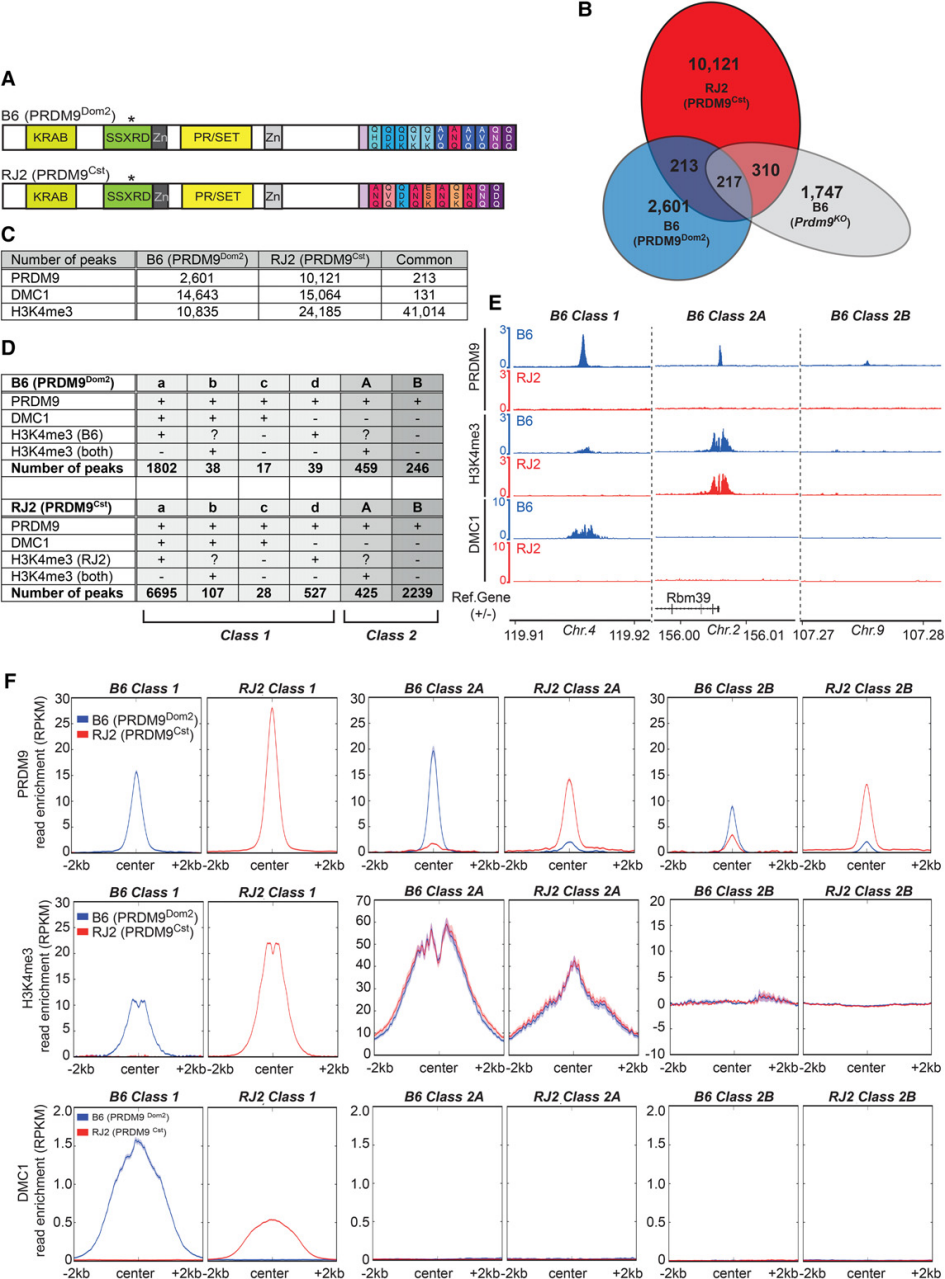
PRDM9 binds to two distinct classes of sites in the genome. (*A*) Schematic representation of the PRDM9^Dom2^ and PRDM9^Cst^ proteins in the B6 and RJ2 mouse strains, respectively. The asterisk indicates the only amino acid substitution outside the zinc finger array due to a nonsynonymous single-nucleotide polymorphism. (*B*) Venn diagram showing the overlap of PRDM9 ChIP-sequencing peaks in B6, RJ2, and B6 *Prdm9*^*KO*^ mice. (*C*) Number of strain specific and common peaks retrieved for PRDM9, DMC1, and H3K4me3 ChIP-seq experiments. (*D*) Classification of PRDM9 peaks in subclasses with (+) or without (−) enrichment for DMC1 and/or H3K4me3 in B6 and RJ2 mice. Question marks indicate situations where a PRDM9-dependent signal (if present) could not be detected because of a common signal also found in the other strain. Class 1 contains PRDM9 peaks that are enriched in DMC1 and/or strain-specific (PRDM9-dependent) H3K4me3. Class 2 sites are negative for DMC1 but can overlap peaks that are enriched for H3K4me3 in both mouse strains. (*E*) Read distribution from PRDM9, H3K4me3, and DMC1 ChIP-seq experiments at representative B6 class 1, 2A, and 2B sites in B6 and RJ2 mice. Read distribution was calculated from pooled replicates, in 1-bp windows, and normalized by library size and input. (*F*) Average read enrichment (reads per kilobase pair per million mapped reads [RPKM]) of PRDM9, H3K4me3, and DMC1 in B6 and RJ2 mice centered on class 1, 2A, and 2B sites in each strain.

To analyze PRDM9 binding to chromatin in optimal conditions, we purified chromatin from testes of prepuberal mice at 13 dpp, where the relative abundance of early stages of meiotic prophase is elevated. We used two mouse strains (C57BL/6, hereafter B6; and RJ2) that harbor *Prdm9* alleles with distinct zinc finger arrays that specify distinct recombination hotspot localizations (Baudat et al. 2010; Grey et al. 2011). The allele present in B6 mice is from *Mus musculus domesticus* (named *Prdm9*^*Dom2*^) and the allele present in RJ2 mice is from *Mus musculus castaneus* (named *Prdm9*^*Cst*^) (Fig. 1A). The genetic background of these two strains is C57BL/6 and C57BL/10, respectively, two nearly identical inbred strains derived from C57BL at the beginning of the 20th century. The anti-PRDM9 antibody used detected a nuclear signal in B6 spermatocytes at leptonema and zygonema that was absent in *Prdm9*^−/−^ B6 spermatocytes (B6 *Prdm9*^*KO*^ hereafter) (Supplemental Figs. S1A, S1B). This antibody recognized efficiently both PRDM9^Dom2^ and PRDM9^Cst^ variants (Supplemental Fig. S1C).

ChIP-seq experiments revealed 3041 and 10,871 PRDM9 binding sites in the B6 and RJ2 strains, respectively (Fig. 1B). The reproducibility between replicates was tested and taken into account using the irreproducible discovery rate (IDR) method (Supplemental Table S1; Supplemental Material). Their specificity was validated by the analysis of B6 *Prdm9*^*KO*^ mice, using nonstringent condition (a relaxed *P*-value) for peak calling, allowing us to remove nonspecific signals (see Supplemental Material). In addition, the few peaks shared between strains (213 peaks), which could be nonspecific, were removed from this pool and analyzed separately, leading to 2601 and 10,121 PRDM9 binding regions specific for the B6 and RJ2 strains, respectively (Fig. 1B). The striking difference in peak numbers between the two strains was not due to a difference in progression in meiosis (Supplemental Fig. S1D). We speculate that the lower number and lower average strength (Supplemental Fig. S2A) of PRDM9^Dom2^ peaks compared with PRDM9^Cst^ is biologically relevant. This could be due to an overall lower affinity of PRDM9^Dom2^ for its binding sites, leading to a reduced occupancy, as detected by ChIP analysis.

PRDM9 binding to DNA is linked to at least two detectable molecular events: H3K4me3 enrichment and DSB formation, which can be monitored by DMC1 association with DSB ends. H3K4me3 and DMC1 profiles have been previously monitored in strains closely related to B6 that express PRDM9^Dom2^ (Smagulova et al. 2011; Brick et al. 2012), as well as the H3K4me3 profile in a mouse strain that expresses a genetically engineered *Prdm9*^*Cst*^ allele in the C57BL/6 background (Baker et al. 2014). Therefore, we mapped DMC1 and H3K4me3 in the B6 and RJ2 strains by ChIP-seq experiments (Supplemental Table S1). We identified about 15,000 DMC1 peaks in each strain that overlap by <1% (14,774 for B6 and 15,195 for RJ2) (Fig. 1C). The similar number of DMC1 peaks in the two strains is in contrast with the lower number and reduced average intensity of PRDM9 peaks in B6 compared with RJ2 testes. H3K4me3 enrichments were quantitatively and qualitatively similar to those reported in previous studies. Specifically, most H3K4me3 sites were shared by both B6 and RJ2 strains (79% of total B6 peaks and 63% of total RJ2 peaks) (Fig. 1C) and significantly overlapped with regulatory elements in the genome (∼60% of common H3K4me3 peaks overlapped with annotated transcription start sites or testes-specific enhancers). Strain-specific H3K4me3 peaks (10,835 for B6 and 24,185 for RJ2) mirrored the site-specific PRDM9 methyltransferase activity, as previously reported (Brick et al. 2012; Baker et al. 2014). The difference in the number of H3K4me3 and DMC1 peaks, compared with PRDM9, could be due to various reasons, for instance, the ChIP assay sensitivity or the shorter half-life of PRDM9 association with its binding sites compared with that of DMC1 or H3K4me3.

Unexpectedly, the comparative analysis of the PRDM9, DMC1, and H3K4me3 sites revealed two strikingly different types of PRDM9 binding sites in both strains. Most binding sites showed the properties expected for PRDM9's role in specifying DSB formation, namely, enrichment for H3K4me3 and DMC1 in addition to PRDM9. We called these sites class 1 sites (73% for both B6 and RJ2). The other binding sites (class 2 sites) showed no detectable DMC1 signal and were enriched (class 2A) or not (class 2B) in PRDM9-independent H3K4me3 (Fig. 1D–F; Supplemental Fig. S2B). Class 2B peaks were on average of lower strength compared with that of class 1 peaks (Supplemental Fig. S2C).

Among class 1 peaks, the average strength of DMC1 was greater in B6 compared with RJ2 (Supplemental Fig. S2D) despite the greater strength of PRDM9 peaks from RJ2 mentioned above (Supplemental Fig. S2A). Among all DMC1 sites, class 1 peaks correspond to the strongest intensity sites, in agreement with our hypothesis that PRDM9 detection is less sensitive than DMC1 detection in our assays (Supplemental Fig. S2E). In class 1 peaks, the average H3K4me3 level was higher in RJ2 than in B6 samples (Fig. 1F), in agreement with the stronger PRDM9 signal observed in RJ2 samples (Fig. 1F; Supplemental Fig. S2A). Overall, we detected a slightly higher correlation between PRDM9 and H3K4me3 than between PRDM9 and DMC1 in both the B6 and RJ2 samples (Supplemental Fig. S2F).

In most class 1 peaks, H3K4me3 enrichment was strain specific, as expected if this histone modification was catalyzed by PRDM9 (Fig. 1D). A small percentage of class 1 peaks (class 1b; 2% for both B6 and RJ2) showed H3K4me3 enrichment in both strains. This could be explained by the presence of an overlapping gene regulatory element (79% and 65% of these class 1b peaks for B6 and for RJ2, respectively, overlapped with a putative promoter or enhancer). This overlap did not allow assessing the presence of PRDM9-dependent H3K4me3 enrichment in these regions. A small fraction of class 1 (class 1d: 2% in B6, 7% in RJ2) peaks did overlap with strain-specific H3K4me3, but not with DMC1. Nevertheless, these sites showed signs of recombination activity (see below).

### PRDM9 binding sites without recombination activity

**Figure 2.**
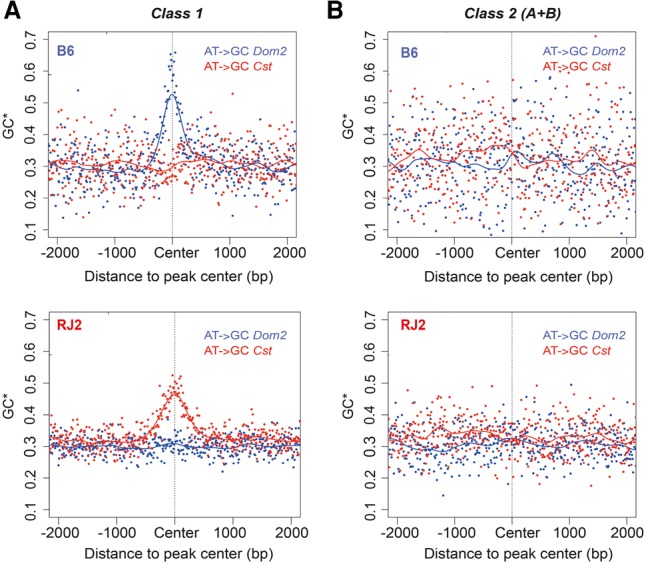
PRDM9 class 1, but not class 2, sites show signatures of GC-biased gene conversion. Points represent the equilibrium GC-content (GC*) estimated from the lineage-specific substitutions aggregated in 10-bp bins from the center of all peaks. GC* in *M. m. domesticus* and *M. m. castaneus* lineages are displayed in blue and red, respectively. Lines were obtained by fitting a cubic smoothing spline. (*A*) GC* centered on PRDM9 class 1 sites in B6 and RJ2 mice. (*B*) GC* centered on PRDM9 class 2 sites in B6 and RJ2 mice; the equilibrium GC-content (GC*) in *M. m. domesticus* and *M. m. castaneus* lineages are not significantly different (Student *P*-value = 0.65 and 0.99 for B6 and RJ2, respectively).

Unexpectedly, class 2 PRDM9 binding sites did not show any detectable DMC1 enrichment (Fig. 1E,F; Supplemental Fig. S2B). Therefore, these sites should not correspond to recombination sites, although a faster DMC1 turnover at these sites cannot be excluded. We thus performed an alternative analysis based on GC content evolution, which is entirely independent from technical and molecular issues. Meiotic recombination activity generates GC-biased gene conversion (Duret and Galtier 2009), and this bias leads to increased rates of A:T-to-G:C substitutions. This effect has been detected at mouse recombination hotspots, defined by DMC1 ChIP-seq (Clement and Arndt 2013). GC-biased gene conversion can be measured by estimating the GC content at equilibrium, also named GC* (see Methods). At class 1 sites, a sharp GC* increase was detected at the center of hotspots, defined by PRDM9 binding, specifically in the lineage where the corresponding PRDM9 allele was present (*M. m. domesticus* for PRDM9^Dom2^, *M. m. castaneus* for PRDM9^Cst^) (Fig. 2A). As expected, GC* level was correlated with PRDM9 peak strength (Supplemental Fig. S3A). Conversely, at class 2 sites (and also classes 2A and 2B separately) (Supplemental Fig. S3B), no GC* increase was observed, indicating the lack of detectable historical recombination activity in these genomic regions (Fig. 2B). The absence of GC* increase in class 2 sites was not due to a limited sample size, because a random subsampling of the same number of class 1 peaks still allowed the detection of GC conversion bias (Supplemental Fig. S3C). The subset of class 1d sites in RJ2 samples (527 sites), although devoid of DMC1 enrichment, also showed statistically significant biased GC gene conversion, indicating that these sites are recombination initiation sites (Supplemental Fig. S3D).

At class 2A sites, H3K4me3 level did not correlate with that of PRDM9, suggesting that this enrichment was catalyzed by other methyltransferases, such as those involved in gene expression regulation (Supplemental Fig. S2G). At these sites, the average H3K4me3 enrichment was higher than that of strain-specific H3K4me3 (Fig. 1F). We thus could not determine whether low PRDM9 methyltransferase activity was present at these class 2A sites.

### H3K36me3 is specifically enriched at recombination sites in a *Spo11*-independent manner

**Figure 3.**
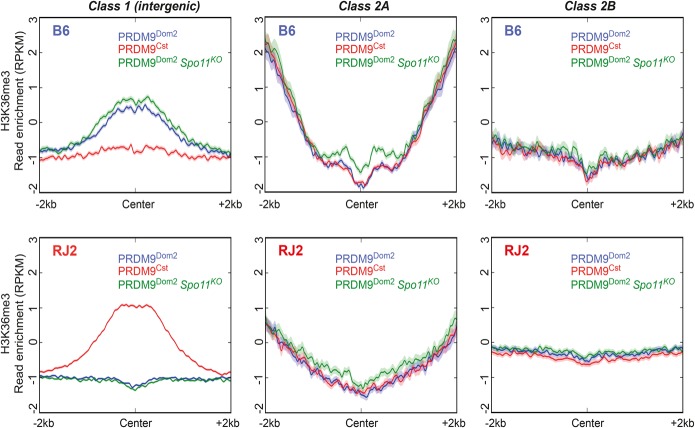
PRDM9-dependent H3K36me3 enrichment in class 1 sites is independent of double-strand break formation. Average and normalized read enrichment (RPKM) of H3K36me3 in B6 (blue), B6 *Spo11*^*KO*^ (green), and RJ2 (red) mice. B6 and B6 *Spo11*^*KO*^ mice have the same PRDM9 allele (*Dom2*). Read enrichments are centered on PRDM9 class 1, 2A, and 2B sites and normalized by subtracting the input read enrichment. Only peaks that overlapped with intergenic regions were considered for class 1 (826 and 3321 sites for B6 and RJ2, respectively) to avoid noise from the H3K36me3 signal specifically found at transcribed genes.

PRDM9 catalyzes H3K36me3 formation in vitro (Wu et al. 2013; Eram et al. 2014), and H3K36me3 enrichment is detected in vivo at the center of mouse hotspots (Davies et al. 2016; Powers et al. 2016). Interestingly, we only detected this PRDM9-dependent H3K36me3 enrichment in class 1 PRDM9 binding sites and not in classes 2A or 2B (Fig. 3).

As class 1 sites are also recombination sites, it was important to determine whether H3K36me3 enrichment was dependent on recombination activity. To this aim, we monitored H3K36me3 distribution on chromatin from B6 *Spo11*^tm1M^ (B6 *Spo11*^*KO*^ hereafter) testes, where initiation of recombination activity is abolished. H3K36me3 level at PRDM9^Dom2^ sites was similar in B6 *Spo11*^*KO*^ and B6 samples (Fig. 3), demonstrating that this modification does not rely on SPO11-dependent DSBs. Therefore, H3K36me3 at recombination sites could be directly catalyzed by PRDM9 methyltransferase activity.

### Epigenetic features of non-DSB sites bound by PRDM9

Class 2 sites were not enriched for H3K36me3 around the PRDM9 binding sites, which is compatible with the absence of PRDM9 methyltransferase activity. However, class 2A sites, which are highly enriched in H3K4me3, were enriched in H3K36me3 in the flanking regions outside the center of the PRDM9 peaks (Fig. 3). This might reflect an overlap of PRDM9 binding sites with promoters and the transcriptional activity of the associated genes. Indeed, in B6 and RJ2 testes, 88% and 72% of class 2A sites, respectively, overlapped with promoters (Supplemental Fig. S4A). To assess histone mark enrichment in class 1, 2A, and 2B sites, we took advantage of available ChIP-seq data for several histone marks in B6 mice: H3K27me3, H3K27ac, and H3K4me1 in spermatocytes from Hammoud et al. (2014); lysine crotonylation (Kcr) in spermatocytes from Tan et al. (2011); and RNA polymerase II (Pol2) in whole testis (Mouse ENCODE Project) (Yue et al. 2014). Class 2A sites from B6 mice and also from RJ2 mice, although to a lesser extent, were strongly enriched in marks associated with active promoters (Supplemental Fig. S4B). The enrichment was centered on the PRDM9 peak. However, class 2B sites were devoid of any significant epigenetic feature. Moreover, genomic features (promoter, genic, and intergenic regions) were stochastically distributed within this class (Supplemental Fig. S4A).

Consistent with the epigenetic marks observed at class 2A sites, these sites were characterized by high CpG density, and most of them overlapped with CpG islands (85% and 53% in B6 and RJ2, respectively). Conversely, class 1 and class 2B sites rarely overlapped with CpG islands (∼1% and ∼6%, respectively). We examined the density of class 2A sites per chromosome and detected a strong positive correlation with CpG islands (Supplemental Fig. S4C). Class 2B sites showed similar patterns, whereas the density in class 1 sites was largely independent of the CpG island content, at the resolution of 1 Mb (Supplemental Fig. S4C). We analyzed the distribution of sites along chromosomes within 10-Mb windows and detected a nonstochastic distribution of PRDM9^Cst^ class 1 sites as shown for Chromosome 1 (Supplemental Fig. S4D). Interestingly, the distribution of PRDM9^Cst^ class 2B sites on Chromosome 1 was similar, and the genome-wide distribution of class 1 sites correlated with that of class 2B sites, reaching a correlation coefficient of 0.71 for 10-Mb window size (Supplemental Fig. S4D). The correlation between PRDM9^Cst^ class 1 and class 2A was weaker, as well as the one observed for the analysis of B6 sites likely due to small sample size (Supplemental Fig. S4D).

### PRDM9 is recruited to non-DSB sites that do not contain its DNA binding motif

**Figure 4.**
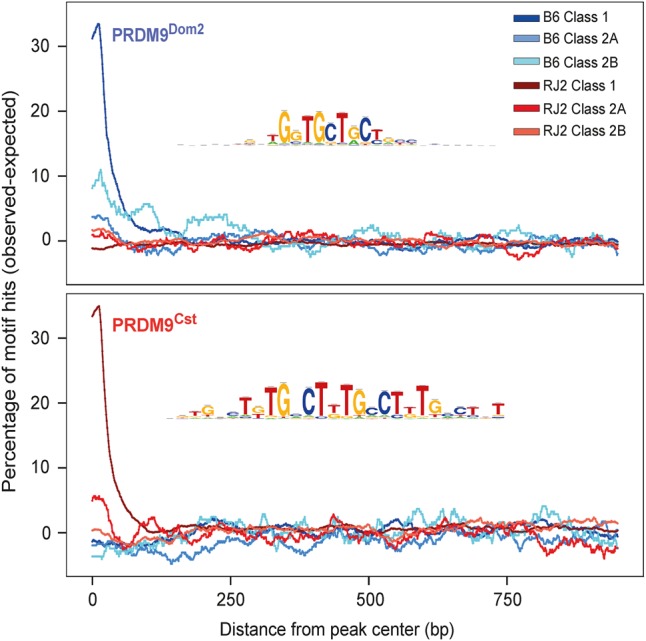
PRDM9 class 1 but not class 2 sites are enriched in PRDM9 allele-specific motifs. Distribution of hits for PRDM9^Dom2^ and PRDM9^Cst^ motifs (each consensus motif is depicted on each graph) along B6 and RJ2 class 1, 2A, and 2B sites from the center of the PRDM9 sites and up to 1 kb of distance. Hits were calculated in a 50-bp sliding window with a 1-bp step.

At mouse DMC1 binding sites, consensus motifs that partially overlap with the in silico prediction of the zinc finger domain specificity for the *Prdm9*^*Dom2*^ allele have been identified (Smagulova et al. 2011; Brick et al. 2012). Similarly, PRDM9^Cst^-specific H3K4me3 sites overlap with a consensus motif for the *Prdm9*^*Cst*^ allele (Baker et al. 2014). Therefore, we asked whether consensus motifs were present in each PRDM9 binding site class. We identified a motif that matched the previously identified motifs (Supplemental Fig. S5A,B) in both PRDM9^Dom2^ and PRDM9^Cst^ class 1 sites, but not in class 2 peak DNA sequences. We then investigated the presence of PRDM9 class 1 consensus motifs in all identified peaks and found that their position overlapped with the center of the PRDM9 peak in class 1 sites (Fig. 4). Conversely, we did not find any significant enrichment for class 1 consensus PRDM9^Dom2^ or PRDM9^Cst^ motifs in class 2A and 2B peaks (Fig. 4). This method was sensitive enough to also detect the presence of PRDM9^Dom2^ or PRDM9^Cst^ motifs in class 1d peaks where the DMC1 signal was undetectable (Supplemental Fig. S5C). These observations strongly suggest that PRDM9 does not bind to class 2 sites through its zinc finger domain or at least not via the zinc fingers involved in recombination site specification. As class 2A sites overlap heavily with promoters, one may speculate that PRDM9 is recruited to these sites by interacting with some transcription machinery components.

PRDM9^Dom2^ class 2B sites were enriched in a motif similar to the one recognized by the insulator protein CTCF (Supplemental Fig. S6A; Nakahashi et al. 2013; Pugacheva et al. 2015). Consistent with this observation, we detected enrichment for the CTCF consensus motif at the center of B6 class 2B sites (Supplemental Fig. S6B). The absolute number of CTCF motif-containing peaks was similar in B6 and RJ2 samples (148 and 259 in B6 and RJ2, respectively, compared with 25 and 38 in randomized controls); however, these sites represented 60% of all class 2B sites in B6 and only 11% in RJ2. By coimmunoprecipitation analysis, we revealed that in vivo, PRDM9 interacts with CTCF in testes. This interaction required the presence of DNA and/or RNA because it was lost upon nuclease treatment (Supplemental Fig. S6C). It seems unlikely that PRDM9 interacts directly with a CTCF binding motif because, using in vitro affinity assays, we did not detect any interaction between PRDM9 zinc fingers and a CTCF consensus motif (F Baudat, pers. comm.). Thus, the interaction between PRDM9 and sites containing the CTCF DNA motif could be the result of an indirect interaction between PRDM9 and CTCF. This interaction could involve soluble PRDM9 or PRDM9 bound to its motif (class 1 sites). Both possibilities are compatible with the recovery of DNA sequences containing CTCF motifs by crosslink ChIP. In RJ2, class 2B represented 23% of all sites compared with 9% in B6. It thus appears that these indirect peaks are recovered more efficiently in RJ2 and that they include, in addition to CTCF sites, a significant number of other genomic sites.

Our analysis also detected a small number of PRDM9 peaks that were common between the two strains B6 and RJ2 (213 peaks) (Fig. 1B; Supplemental Fig. S7A,B) but absent in *Prdm9*^*KO*^ and that could be sites where PRDM9 binding is not directed by its DNA sequence specificity. Based on DMC1 and H3K4me3 analysis, 27% and 60% of these peaks correspond to classes 2A and 2B, respectively (Supplemental Fig. S7C). Motif search showed the absence of motif enrichment for PRDM9^Dom2^, PRDM9^Cst^, and CTCF (Supplemental Fig. S7D).

### PRDM9 binding is *Spo11* dependent at class 2A, but not at class 1 and class 2B sites

**Figure 5.**
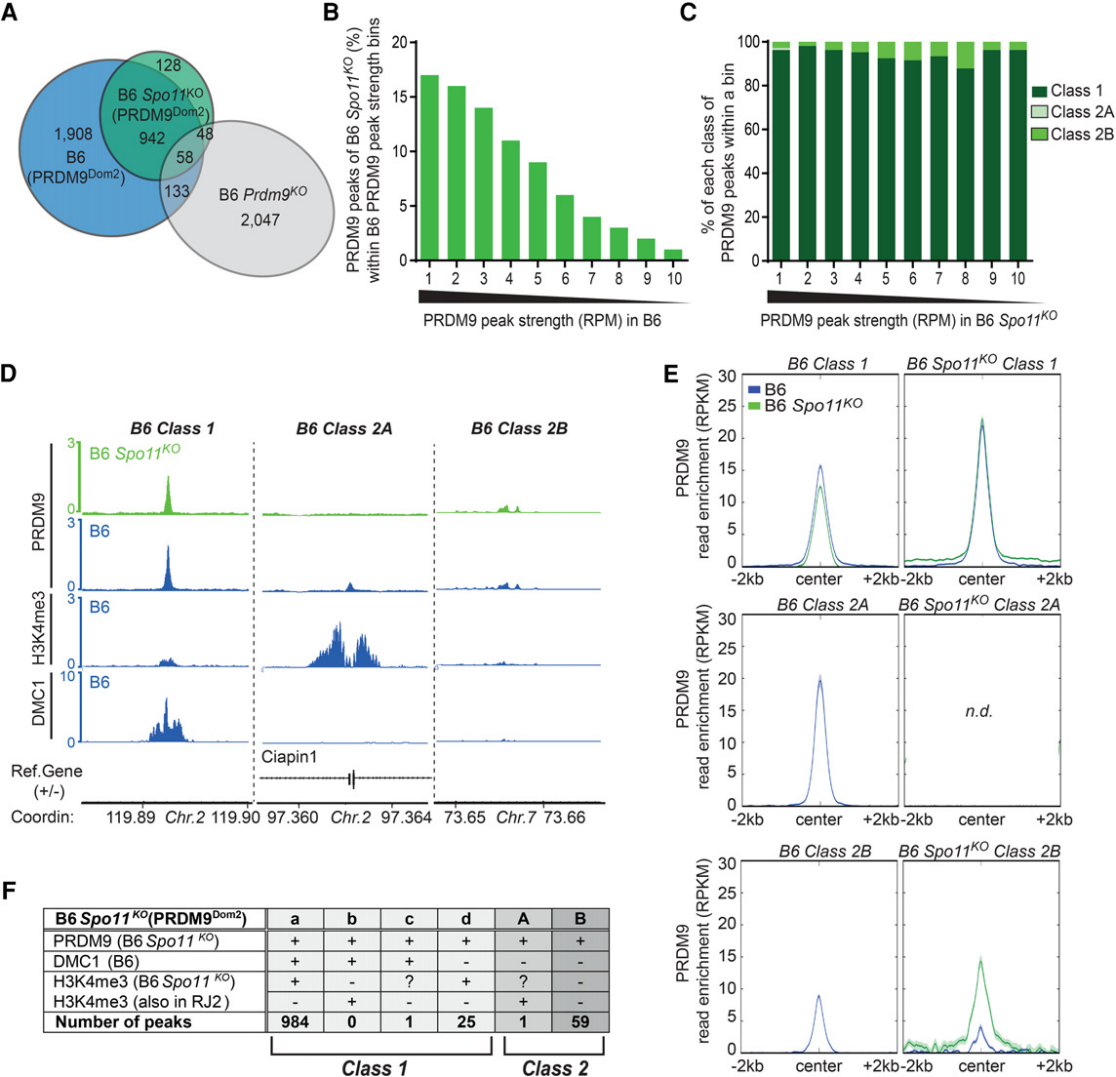
Class 2A, but not class 1 or class 2B, sites are *Spo11* dependent. (*A*) Venn diagram showing the overlap of PRDM9 ChIP-seq peaks from B6 (all 3041 specific and common peaks), B6 *Spo11*^*KO*^ (PRDM9^Dom2^), and B6 *Prdm9*^*KO*^ mice. (*B*) PRDM9 B6 peaks (*Spo11*^+/+^) were binned by strength. In each bin, the percentage of B6 *Spo11*^*KO*^ (PRDM9^Dom2^) overlapping peaks was plotted. (*C*) Distribution of classes into bins of PRDM9 peak strength monitored in B6 *Spo11*^*KO*^ mice. (*D*) Read distribution from PRDM9 ChIP-seq at representative class 1, 2A, and 2B sites found in B6 *Spo11*^*KO*^ mice compared with the read distribution from PRDM9, H3K4me3, and DMC1 ChIP-seq in B6 mice at the same sites. Read distribution was calculated from pooled replicates, in 1-bp windows, and normalized by library size and input. (*E*) Average read enrichment (RPKM) of PRDM9, in B6 and B6 *Spo11*^*KO*^ mice centered on class 1, 2A, and 2B sites of each strain. (n.d.) Not determined (only one class 2A peak was identified in B6 *Spo11*^*KO*^). (*F*) Classification of the PRDM9-positive peaks detected in B6 *Spo11*^*KO*^ mice in subclasses with (+) or without (−) enrichment for DMC1 (measured in B6 mice) and/or H3K4me3 (specifically present in B6 *Spo11*^*KO*^ mice or also present in RJ2). Classes were defined as in Figure 1D. H3K4me3 ChIP-seq data for B6 *Spo11*^*KO*^ (PRDM9^Dom2^) are from Brick et al. (2012) and downloaded from the GEO database (GSE35498).

SPO11 is responsible for the formation of meiotic DSBs during the leptotene stage in mice (Baudat et al. 2000; Romanienko and Camerini-Otero 2000). On the other hand, PRDM9-dependent H3K4me3/H3K36me3 enrichment at recombination hotspots does not require *Spo11* (Fig. 3; Buard et al. 2009). Thus, one could hypothesize that PRDM9 binding to DSB sites occurs prior to DSB formation, independently of *Spo11*. Conversely, PRDM9 recruitment to all or some of the noncanonical binding sites might depend on DSB formation catalyzed by SPO11. To evaluate the relationship between the different classes of PRDM9 binding sites and recombination initiation, we monitored PRDM9 binding in B6 *Spo11*^*KO*^ testes. We detected 1070 peaks (after filtering out the nonspecific signal that overlapped with B6 *Prdm9*^*KO*^), 88% of which (*n* = 942) overlapped with those mapped in B6 (Fig. 5A), and validated by analysis of the two replicates (see Methods). As B6 *Spo11*^*KO*^ peaks overlapped largely with strong intensity B6 peaks, their lower number could reflect a lower sensitivity of this experiment (Fig. 5B). These peaks could be classified into the three classes of 1, 2A, and 2B, similarly to the analysis of B6 mice (Fig. 5C–F; Supplemental Fig. S8). The peak distribution in the different classes differed, however, significantly between the B6 and B6 *Spo11*^*KO*^ samples (χ^2^ = 272.26, df = 2, *P*-value <10^−6^). Specifically, class 2A peaks were almost completely absent in B6 *Spo11*^*KO*^ samples (1010 class 1, 1 class 2A, and 59 class 2B sites in B6 *Spo11*^*KO*^, respectively, compared with 1896, 459, and 246 sites in B6) (Figs. 1D, 5F). This difference was not due to the bias toward stronger sites. Indeed, the distribution of the 1070 strongest peaks in B6 (861 class 1, 198 class 2A, and 11 class 2B sites, respectively) was significantly different compared with that of the B6 *Spo11*^*KO*^ peaks (χ^2^ = 431.24, df = 2, *P*-value <10^−6^). We conclude that class 2A sites are *Spo11* dependent. Conversely, class 2B sites in B6 *Spo11*^*KO*^ samples shared the same property as class 2B sites in B6 samples, indicating that these potential indirect interactions between PRDM9 and other genomic sites do not require SPO11.

## Discussion

### Direct evidence of PRDM9 binding at recombination hotspots

We report here the first extensive analysis of the in vivo binding of PRDM9 in two mouse strains that express PRDM9 variants with different zinc finger arrays. We show that PRDM9 binds to two classes of sites: sites at recombination hotspots (class 1) and sites that do not show any sign of recombination (class 2). We also demonstrate that PRDM9 binding to recombination hotspots (class 1 sites) is *Spo11* independent.

The detection of a higher number of PRDM9 binding sites and, in average, a greater strength in the strain expressing PRDM9^Cst^ compared with the one expressing PRDM9^Dom2^, in a *M. m. domesticus* genetic background, suggests interesting differences in PRDM9 properties according to the genetic context. It is unlikely that these very substantial differences in binding site number and strength are due to technical artefacts given the many controls included in the experiments. These differences may reflect a lower in vivo occupancy of PRDM9^Dom2^ motifs compared with PRDM9^Cst^ motifs due to differential affinities of the proteins for their binding sites. However, in vitro binding assays of PRDM9 to DNA sequences from strong hotspots did not reveal any major affinity difference between these zinc finger domains (F Baudat, pers. comm.). This suggests the alternative interpretation that the sites available for PRDM9 binding show, on average, lower affinity for PRDM9^Dom2^ compared to PRDM9^Cst^ in the B6 genome. This effect could be caused by erosion of PRDM9^Dom2^ motifs by gene conversion (Myers et al. 2010; Baker et al. 2015). As the PRDM9^Dom2^ protein is produced in the *M. m. domesticus* C57BL/6 strain and PRDM9^Cst^ is a variant derived from *M. m. castaneus* and not found in *M. m. domesticus* (Buard et al. 2014; Kono et al. 2014), erosion should have, indeed, affected only the PRDM9^Dom2^ sites on the B6 genome present in the analyzed strains.

The level of DMC1 activity (number of sites and reads) was not lower in B6 compared with RJ2 as measured by ChIP-seq. This shows that PRDM9 binding in the B6 strain is not limiting in these conditions and suggests that additional factors that act downstream from PRDM9 binding regulate DSB activity. This is an important feature of DSB regulation that may, in part, explain why hotspots, which were previously identified as sharing the same PRDM9 motif, could have distinct recombination activities (Berg et al. 2011). Mechanistically, one could propose that any step after PRDM9 binding, such as SPO11 recruitment and/or activation, could contribute to this effect.

### Histone modifications associated with PRDM9 binding: a role for DSB activation?

The presence of H3K4me3 and H3K36me3 has been detected at nucleosomes around the center of mouse hotspots; H3K4me3, around the center of human hotspots (Fig. 3; Buard et al. 2009; Grey et al. 2011; Smagulova et al. 2011; Pratto et al. 2014; Davies et al. 2016; Powers et al. 2016). These studies showed that these modifications are PRDM9 dependent and thus are predicted to be catalyzed by PRDM9. Our direct evidence of PRDM9 binding at these sites further supports this hypothesis. Also, as previously shown for H3K4me3 (Buard et al. 2009; Grey et al. 2011), we found here that H3K36me3 at hotspots is not a downstream consequence of DSB formation as it is detected in B6 *Spo11*^*KO*^ mice. H3K36me3 may play a role in specifying DSB formation and/or in DSB repair, as described in somatic cells (Fnu et al. 2011; Carvalho et al. 2014; Pai et al. 2014; Pfister et al. 2014). In somatic cells, H3K36me3 favors DSB repair by homologous recombination through regulation of end processing (Clouaire and Legube 2015), and meiotic DSB repair is specifically channeled toward the homologous recombination pathway (Hunter 2015).

### PRDM9 interacts with a subset of its genomic targets independently of its zinc finger specificity

In class 2 sites, the absence of detectable motifs that share similarity with PRDM9 consensus sequences strongly suggests that the PRDM9 zinc finger array does not interact with DNA, at least not in the canonical way. As these sites do not show the expected features of PRDM9 binding, it was important to exclude the possibility that they may result from technical artefacts. This was done by removing from our analysis peaks that were present in B6 *Prdm9*^*KO*^, as well as peaks common to B6 and RJ2, which could potentially be false positives. The presence of some false-positive peaks, corresponding to recognition of another protein by our anti-PRDM9 antibody, among those selected cannot be formally excluded. This would, however, imply the rather unlikely possibility that their detection is reproducible in the same genotype but not between the two strains. Furthermore, a large fraction of these sites (class 2B) do not overlap with regions of accessible chromatin that were considered as artifacts in some ChIP-seq analyses (Jain et al. 2015). Conversely, class 2A sites, which mainly overlap with promoters and thus with accessible chromatin, were not detected systematically in all experiments as they were absent in B6 *Spo11*^*KO*^.

**Figure 6.**
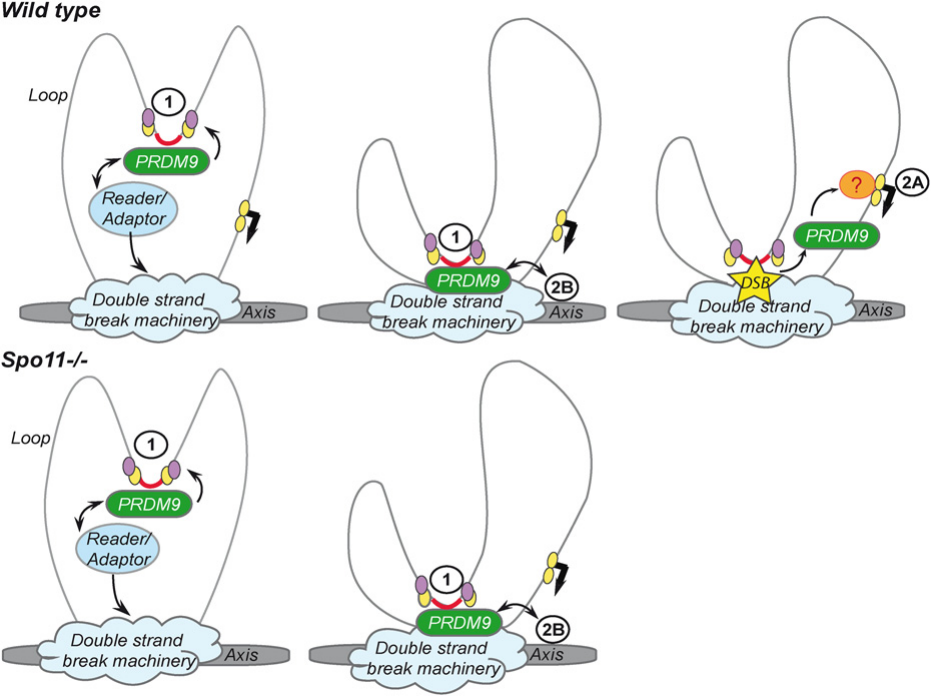
Model of PRDM9 binding dynamics in mouse testes. In early meiotic prophase, when PRDM9 is expressed, chromosomes are predicted to be organized in a characteristic loop-axis structure, shaped by cohesins and other proteins. Some essential components of meiotic DSB formation, such as MEI4 and IHO1, are located as discrete foci on the axis. In wild-type animals, PRDM9, through its zinc finger domain, binds to class 1 DNA motifs (red) that are meiotic recombination hotspots and located in chromatin loops. PRDM9 modifies the surrounding nucleosomes by promoting H3K4me3 (yellow) and H3K36me3 (purple) deposition. Then, a reader or adaptor protein promotes PRDM9 interaction with the DNA double-strand break formation machinery located on the axis. Therefore, PRDM9 can also indirectly interact with DNA sequences near or on the axis. We call these sites PRDM9 class 2B binding sites. SPO11 may be recruited at PRDM9 binding sites before or after the loop-axis interaction. Upon DSB formation, PRDM9 could be displaced and interact with other sites (class 2A that are mainly transcription start sites), possibly through interaction with other unknown factors (question mark). Class 2 sites differ in mouse strains with different PRDM9 alleles. The selection of class 2 sites may depend on their spatial proximity to class 1 sites. In mice deficient for SPO11 (*Spo11*^−/−^ or *Spo11*^*KO*^), PRDM9 binds to class 1 sites and is brought close to the axis, leading to interactions detected as class 2B sites. However, in the absence of SPO11, PRDM9 is not competent or not released in a timely manner to bind to class 2A sites.

Interestingly, PRDM9 class 2 sites differ in the RJ2 and B6 strains but contain no identified PRDM9 allele-specific binding motif. Therefore, we hypothesize that their location is somehow indirectly specified by the binding of PRDM9 with class 1 sites that is determined by the PRDM9 zinc finger array. For instance, proximity with a class 1 site could be a necessary feature for class 2 site interaction with PRDM9, and this interaction might, consequently, involve motif-bound PRDM9 rather than soluble PRDM9 (Fig. 6). We tested this hypothesis; however, we did not detect specific proximity (in base pairs) between class 1 (or DMC1 peaks) and class 2 sites. Therefore, additional factors could contribute to the selection of class 2 sites by PRDM9 bound to class 1 sites such as the three-dimensional chromatin organization. Whatever the mode of PRDM9 recruitment to these sites, we can conclude that PRDM9 interaction with these class 2 sites does not provide a proper context for DSB formation as no recombination could be detected at these sites.

### PRDM9 is recruited to promoters in a *Spo11*-dependent manner

Class 2A sites are mainly promoters that are mostly active at the beginning of meiotic prophase (99% are expressed in spermatocytes at leptotene/zygotene) (da Cruz et al. 2016). Their *Spo11* dependency could imply that DSB formation or progression throughout meiotic prophase is required for PRDM9 interaction with these sites. DSBs are induced within and around PRDM9 binding sites (Lange et al. 2016), and this could result in *Spo11*-dependent PRDM9 displacement. We suggest that this displacement leads to PRDM9 interaction with promoters. As mentioned above, we propose that the location of the promoter with which PRDM9 interacts depends on the location of the DSB sites. The molecular nature of this dependency remains to be determined (Fig. 6). It would also be interesting to determine whether PRDM9 is recruited to class 2A sites through interaction with some transcription machinery components at these promoters. Whatever the mechanism leading to these alternative interactions, it also raises the question of their function. They could be a “by-product” of PRDM9 activity as a member of the PRDM family of transcription regulators (Fog et al. 2012), but they might also have a specific, not yet determined function.

### PRDM9 sites compatible with loop-axis interactions

Class 2B sites combine the unexpected properties of PRDM9 binding in a DSB-independent manner without showing the expected consequences of this binding, namely, H3K4me3 and recombination, based on the DMC1 and GC* analyses. The mechanism of PRDM9 interaction with class 2B sites remains to be investigated. CTCF could be involved in PRDM9 interaction with some class 2B sites. We detected the CTCF binding motif in 60% of class 2B sites in the strain expressing PRDM9^Dom2^. In our coimmunoprecipitation assays, the PRDM9–CTCF interaction is sensitive to benzonase (Supplemental Fig. S6C). Therefore, it is more likely that this interaction is indirect and involves DNA or RNA. CTCF is an insulator protein that binds to promoters and enhancers, at the border of topologically associated domains (TADs), and its binding sites partially overlap with cohesin binding sites (Ong and Corces 2014). Given the cohesin enrichment along meiotic chromosome axes at the stage of DSB formation (McNicoll et al. 2013), we speculate that a fraction of CTCF could be axis-associated, as reported in somatic cells (Tedeschi et al. 2013). PRDM9 could thus interact indirectly with DNA sequences located on the axis, and the allele specificity of these sites favors a model where PRDM9 molecules bound to class 1 sites are involved in these interactions, as discussed above. An example of such indirect interactions involves the insulator protein BEAF-32 (Liang et al. 2014). According to this model, the class 2B signal uncovered by our analysis reveals an interaction between two genomic loci: one DSB site and one axis-associated region (Fig. 6). Other genomic sites that do not include CTCF motifs are recovered through these proposed indirect interactions, particularly in the presence of PRDM9^Cst^. It will be interesting to test whether they are associated with other axis proteins. In the RJ2 strain where the number of sites was the greatest, we could detect a strong correlation between the distribution of class 2B and class 1 sites over 5- to 10-Mbp intervals. This correlation is compatible with a loop-axis interaction involved in PRDM9 binding to these sites, which will lead to spatial constraints between class 1 and 2B sites (Supplemental Fig. S4D). The evidence for loop-axis interaction in *S. cerevisiae* and its role on DSB formation was based on the mapping of axis and DSB sites, and the observation that several proteins essential for DSB formation (Rec114, Mei4, and Mer2, the RMM complex) and DSB repair are located on the chromosome axis. These RMM proteins, as well as axis proteins important for their localization (Hop1 and Red1), are not evenly distributed along chromosomes leading to domains with high or low meiotic DSB activity (Blat et al. 2002; Panizza et al. 2011). It is interesting to note that PRDM9 peak density is not evenly distributed along mouse chromosomes, and it will be interesting to evaluate whether it is related to features of chromosome organization. Two proteins required for meiotic DSB formation in mice (MEI4 and IHO1) are also located on the chromosomes axis (Kumar et al. 2010, 2015; Stanzione et al. 2016) and may also be unevenly distributed along chromosomes.

In conclusion, by monitoring PRDM9 binding in vivo, we provide unprecedented insights into the molecular interactions associated with meiotic recombination hotspot activity. Our approach highlights the negative effect of hotspot erosion on PRDM9 affinity for DNA. The important level of hotspot erosion in the *M. m. domesticus* C57BL/6 strains suggests that the *Prdm9*^*Dom2*^ allele has been active for many generations in this subspecies. As several steps are needed from PRDM9 binding to DSB formation, it is not surprising that the two events are only partially correlated. This brings interesting questions concerning the additional elements involved, which could include interactions with axis proteins that are predicted to be required for activation of the SPO11/TOPOVIBL complex. Our study provides the first analysis in the mouse suggesting that DSB sites interact with other, possibly axis-associated, genomic regions. Remarkably, we also discovered a new category of PRDM9 binding sites, where PRDM9 binding is *Spo11*-dependent. This finding outlines another challenge concerning the analysis of PRDM9 interaction dynamics during DSB formation and repair.

## Methods

### Mouse strains

The following mouse strains were used: C57BL/6JOlaHsd (B6), B10.MOLSGR(A)-(D17Mit58-D17Jcs11)/Bdm (RJ2) (Grey et al. 2009), B6;129P2-Prdm9^tm1Ymat^/J (B6 *Prdm9*^*KO*^) (Hayashi et al. 2005), and *Spo11*^*tm1M*^. This *Spo11*^*KO/KO*^ strain (hereafter B6 *Spo11*^*KO*^) carries the *Prdm9*^*Dom2*^ allele from the B6 strain (Baudat et al. 2000). RJ2 has a C57BL/10 genetic background, which is highly similar to B6, and expresses *Prdm9*^*Cst*^ (previously named *Prdm9*^*wm7*^). B6 and B6 *Spo11*^*KO*^ express *Prdm9*^*Dom2*^ (previously named *Prdm9*^*b*^). All animal experiments were carried out according to the CNRS guidelines and approved by the ethics committee on live animals (project CE-LR-0812 and 1295).

### Antibodies

Rabbit polyclonal antibodies against PRDM9 were developed as described in the Supplemental Methods. A list of the other antibodies used in this article is in the Supplemental Methods.

### ChIP-seq experiments and analysis

For H3K4me3 and DMC1 ChIP-seq experiments, we used the protocols described by Buard et al. (2009) and Khil et al. (2012), respectively.

Details on PRDM9 and H3K36me3 ChIP-seq experiments are described in the Supplemental Methods. Numbers of uniquely mapped reads are shown in Supplemental Table S1.

### Computational data analysis

For ChIP-seq data processing, reads were mapped to the UCSC build mm9 mouse genome assembly. Peaks were identified from unique and high-quality mapped reads. Detailed procedure and parameters of peak calling and downstream analysis are given in the Supplemental Methods.

### GC-biased gene conversion signature analysis

Substitutions that occurred in the *M. m. castaneus* lineage or in the *M. m. domesticus* lineage were identified by comparison with the *Mus spretus* outgroup. Details are given in the Supplemental Methods.

### Immunostaining

Immunostaining experiments using histological sections and chromosome spreads are detailed in the Supplemental Methods.

### PRDM9 protein extraction, immunoprecipitation, and Western blotting

Nuclear extracts were prepared as described earlier (Dignam et al. 1983). Details for immunoprecipitation and Western blot procedures are described in the Supplemental Methods.

## Data access

The raw and processed sequencing files produced in this study (ChIP-seq data listed in Supplemental Table S1) have been submitted to the NCBI Gene Expression Omnibus (GEO; http://www.ncbi.nlm.nih.gov/geo/) under accession number GSE93955.

## Supplementary Material

Supplemental Material
