# Supplementary material for: In vivo binding of PRDM9 reveals interactions with noncanonical genomic sites
Source: Genome Res. 2017 Apr;27(4):580–90. doi: 10.1101/gr.217240.116 (PMC5378176; doi:10.1101/gr.217240.116)
Supplement: Supplemental Material [file supp_27_4_580__index.html]

In vivo binding of PRDM9 reveals interactions with noncanonical genomic sites — Supplemental Material 

# In vivo binding of PRDM9 reveals interactions with noncanonical genomic sites

## Supplemental Material

- Supplemental\_Fig\_S1.pdf
- Supplemental\_Fig\_S2.pdf
- Supplemental\_Fig\_S3.pdf
- Supplemental\_Fig\_S4.pdf
- Supplemental\_Fig\_S5.pdf
- Supplemental\_Fig\_S6.pdf
- Supplemental\_Fig\_S7.pdf
- Supplemental\_Fig\_S8.pdf
- Supplemental\_Fig\_S9.pdf
- Supplemental\_Table\_S1.pdf
- Supplemental\_Information.docx
