# Supplementary material for: In vivo binding of PRDM9 reveals interactions with noncanonical genomic sites
Source: Genome Res. 2017 Apr;27(4):580–90. doi: 10.1101/gr.217240.116 (PMC5378176; doi:10.1101/gr.217240.116)
Supplement: Supplemental Material [file supp_gr.217240.116_Supplemental_Fig_S1.pdf]

Supplemental Figure 1

A

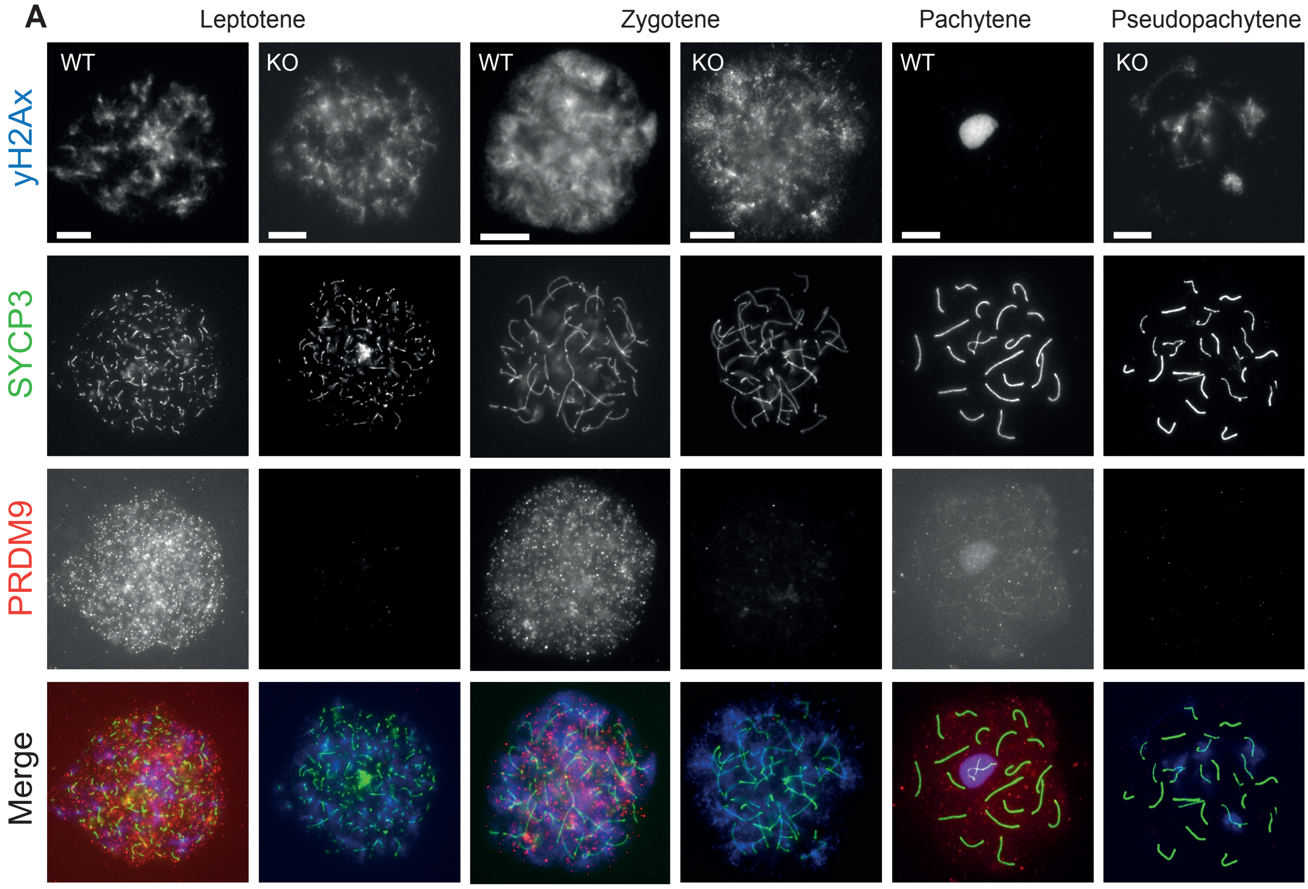

B

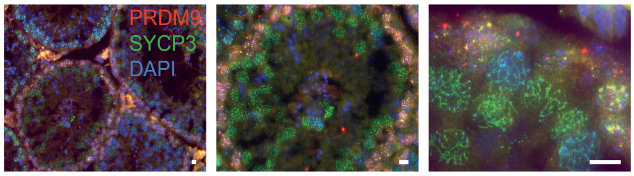

C

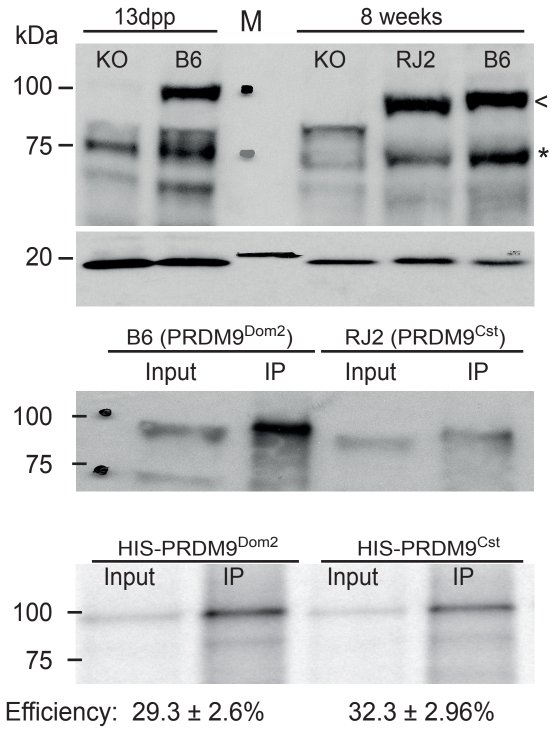

D

	**B6 (PRDM9^{Dom2}) n=2**	**RJ2 (PRDM9^{Cst}) n=2**
Pre-Leptotene	218	156
Leptotene	78	93
Early/Mid Zygotene	52	89
Late Zygotene	21	50
Pachytene	21	37
Total SYCP3 positive nuclei	390	425

Chi-square B6 vs RJ2 p-val: 0.96

Supplemental Figure S1 Anti-PRDM9 antibody binds specifically and with the same efficiency in both B6 and RJ2 mouse strains. (A) Immunofluorescence staining on adult mouse spermatocyte spreads. In B6 wild type, PRDM9 staining appears in leptotene and disappears in pachytene spermatocytes. B6 $PRDM9^{KO}$ mouse spermatocytes do not stain. Nuclei are also labelled with anti-SYCP3 and anti-γH2AX antibodies. (B) Immunofluorescence staining on cryosections of adult B6 testes. PRDM9 antibody stains the outer layer of seminiferous tubules corresponding to leptotene and zygotene spermatocytes. Sections are stained with DAPI and labelled with anti-SYCP3 antibody. (C) Western blot and immunoprecipitation of PRDM9 extracted from adult or juvenile testes or of recombinant PRDM9. Upper panel: Western blot of PRDM9 extracted from juvenile (left, 13dpp) and adult (right, 8 weeks) testis extracts. $PRDM9^{Dom2}$ (B6) migrates with an apparent molecular weight of approximately 100kDa. A slight shift of migration was observed for $PRDM9^{Cst}$ (RJ2), which has one zinc finger less than $PRDM9^{Dom2}$. (*) marks a band which could be due to PRDM9 degradation. KO testis extracts also have some bands, notably one around 75kDa, which could be due to non-specific detection or detection of a truncated protein expressed in B6 $PRDM9^{KO}$ mice. Loading control: Histone H3. Middle panel: Immunoprecipitation of PRDM9 in testes, for inputs 70µg of extract was loaded and for IPs the equivalent of 130µg of immunoprecipitated extract was loaded. Lower panel: Immunoprecipitation of radiolabelled *in vitro*-translated His-PRDM9 *Dom2* and *Cst*. The efficiency of immunoprecipitation is shown +/- SE (standard error of the mean, n=3). (D) Staging of SYCP3 positive nuclei in 12.5dpp spermatocytes in B6 and RJ2 mice. SYCP1, SYCP3 and γH2Ax were used to identify prophase I sub-stages (see Supplemental Methods) (n, number of mice).
