# Supplementary material for: In vivo binding of PRDM9 reveals interactions with noncanonical genomic sites
Source: Genome Res. 2017 Apr;27(4):580–90. doi: 10.1101/gr.217240.116 (PMC5378176; doi:10.1101/gr.217240.116)
Supplement: Supplemental Material [file supp_gr.217240.116_Supplemental_Fig_S2.pdf]

Supplemental Figure 2

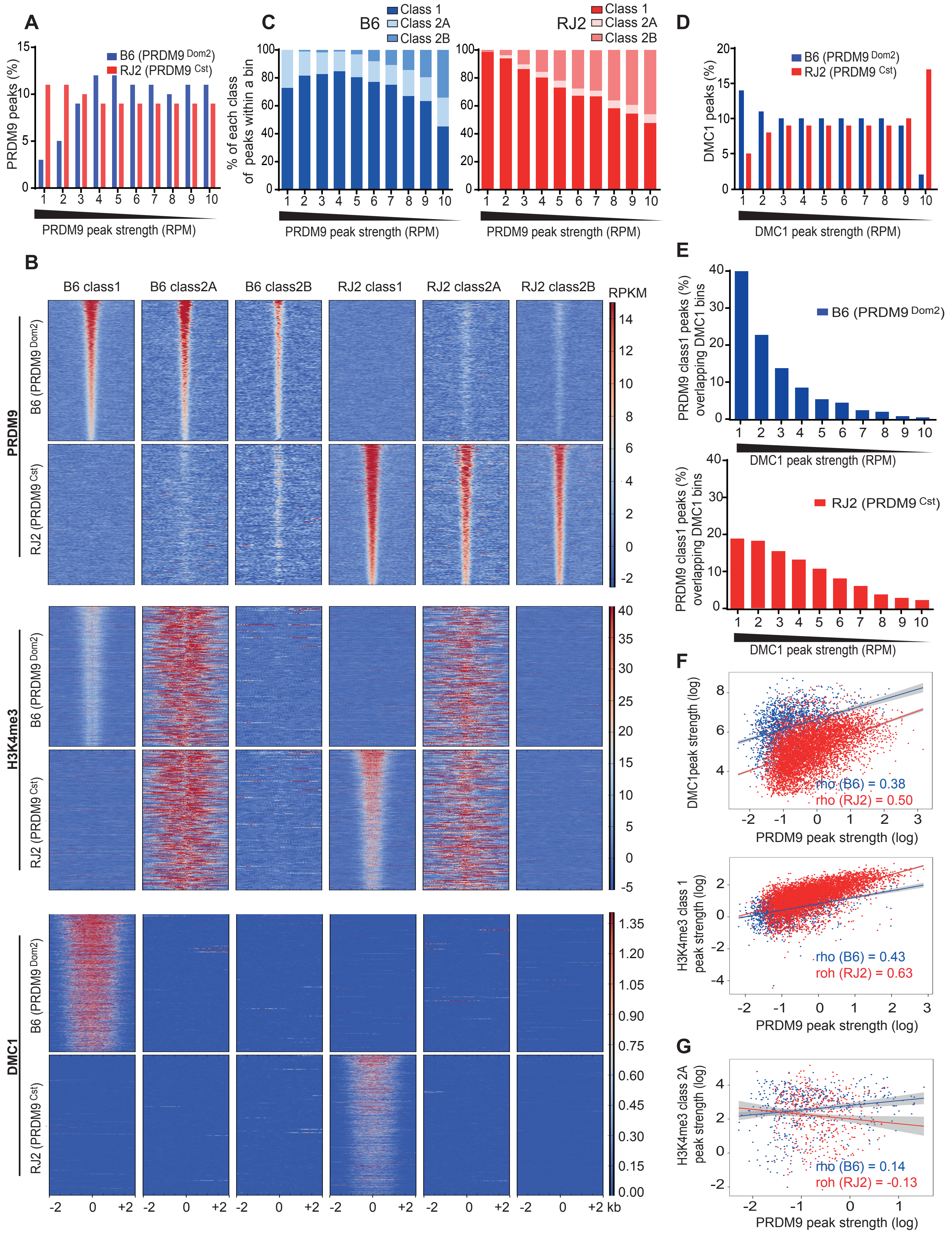

Supplemental Figure S2 PRDM9 class 2 sites lack DSBs and PRDM9-dependent H3K4me3. (A) PRDM9 B6 and RJ2 peaks are pooled and binned by strength (x-axis). Bin 1 representing the strongest peaks and bin 10 the weakest, based on read enrichment. The percentage of PRDM9 B6 or RJ2 peaks overlapping each bin is plotted. (B) Heatmaps of PRDM9, H3K4me3 and DMC1 reads distribution centered on class1, 2A and 2B PRDM9 peaks in B6 and RJ2 mice. (C) Distribution of B6 or RJ2 PRDM9 classes into bins of PRDM9 B6 (left panel) or RJ2 (right panel) peak strength (1 is the strongest and 10 the lowest). (D) DMC1 B6 and RJ2 peaks (all DMC1 peaks) are pooled and binned by strength (x-axis). Bin 1 representing the strongest peaks and bin 10 the weakest, based on read enrichment. The percentage of DMC1 B6 or RJ2 peaks in each bin is plotted. (E) DMC1 B6 and RJ2 peaks are binned by strength (x-axis). Bin 1 represents the peaks with the highest number of reads and bin 10 with the lowest. In each bin the percentage of PRDM9 class 1 peaks is plotted (B6: upper panel and RJ2: lower panel). (F) Scatter plots of PRDM9 strength (log(RPM)) versus DMC1 or H3K4me3 strength . Top: B6 and RJ2 PRDM9 class 1 peak strength versus DMC1 peak strength. Bottom: B6 and RJ2 PRDM9 class 1 peak strength versus H3K4me3 PRDM9-dependent (class 1) peak strength. (G) Scatter plot of PRDM9 strength (log(RPM)) versus H3K4me3 strength at B6 and RJ2 PRDM9 class 2A sites. Rho is the Spearman correlation coefficient.
