# Supplementary material for: In vivo binding of PRDM9 reveals interactions with noncanonical genomic sites
Source: Genome Res. 2017 Apr;27(4):580–90. doi: 10.1101/gr.217240.116 (PMC5378176; doi:10.1101/gr.217240.116)
Supplement: Supplemental Material [file supp_gr.217240.116_Supplemental_Fig_S3.pdf]

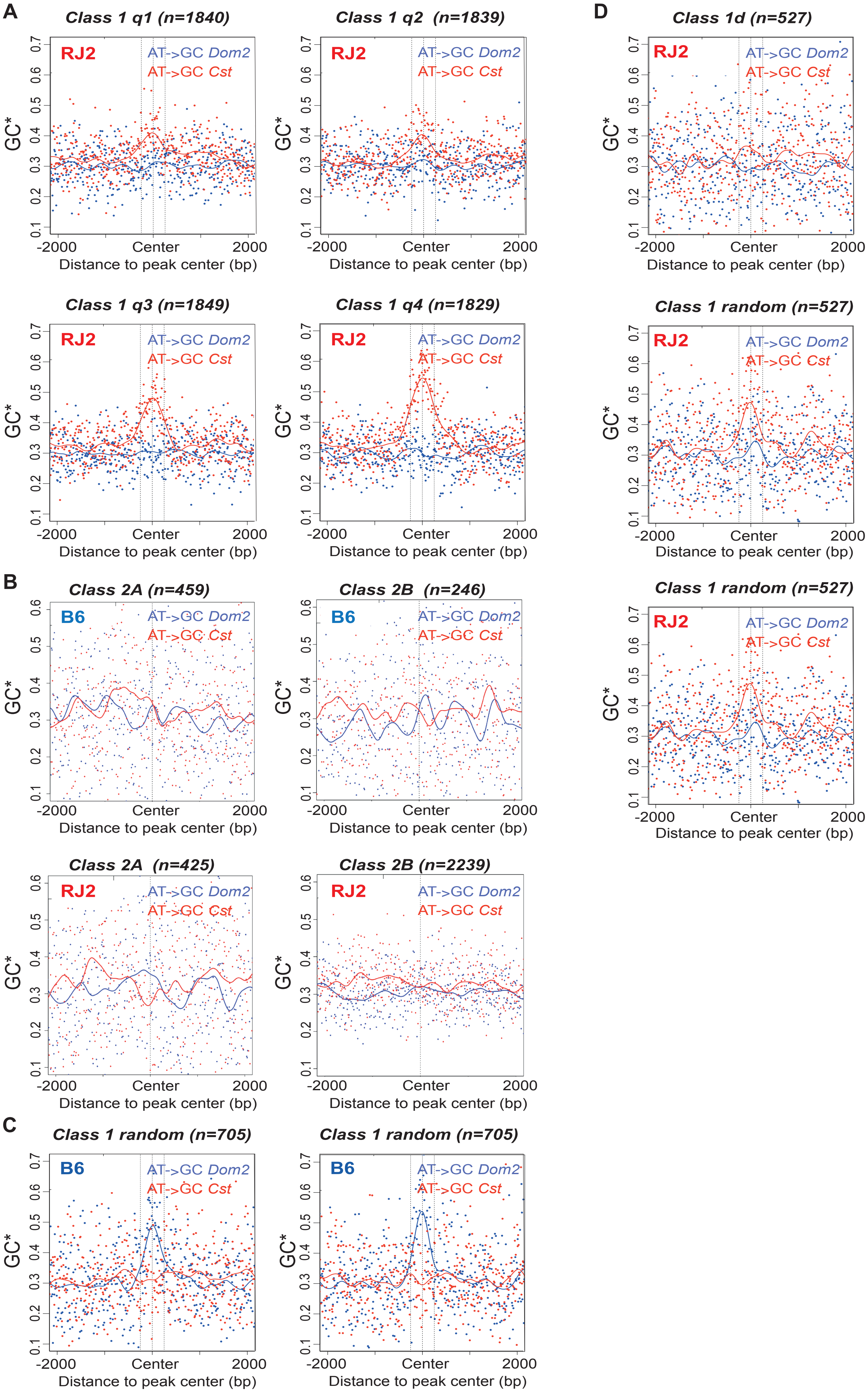
A
Class 1 q1 (n=1840)
Class 1 q2 (n=1839)
Class 1 q3 (n=1849)
Class 1 q4 (n=1829)
RJ2
AT->GC Dom2
AT->GC Cst
GC*
0.1
0.2
0.3
0.4
0.5
0.6
0.7
-2000
Center
2000
Distance to peak center (bp)
B
Class 2A (n=459)
Class 2B (n=246)
B6
Class 2A (n=425)
Class 2B (n=2239)
C
Class 1 random (n=705)
Class 1 random (n=705)
D
Class 1d (n=527)
Class 1 random (n=527)
Class 1 random (n=527)

Supplemental Figure S3 Signature of GC biased gene conversion in RJ2 class 1 sites, control samples of class 1 sites and class 1d sites (DMC1 negative peaks). (A, B) Signature of GC biased gene conversion. Points represent the equilibrium GC-content (GC*) estimated from the lineage-specific substitutions aggregated in 10-bp bins from the center of all peaks. GC* in *M. m. domesticus* and *M. m. castaneus* lineages are displayed in blue and red respectively. Lines were obtained by fitting a cubic smoothing spline. (A) RJ2 peaks are split in four quartiles (q) of peak strength, q1 are the weakest RJ2 class 1 sites and q4 the strongest. (B) Class 2A and 2B peaks do not show significant GC biased gene conversion (center ± 250bp). Student test p-values: B6 Class 2A: 0.4, B6 Class 2B: 0.7, RJ2 Class 2A: 0.03, RJ2 Class 2B: 0.5 (C) Random sub-samples of PRDM9 class 1 sites. The two panels were computed using two independent random sub-samples of PRDM9 B6 class 1 sites (N=705). (D) Average enrichment of GC content equilibrium (GC*) in DMC1 negative RJ2 class1 sites (class 1d; N=527) (top) and two control sets of the same number of peaks from randomly chosen RJ2 class 1 DMC1-positive peaks (middle and bottom). In RJ2 class 1d sites, although the signal is noisier, the average GC* within peaks (center ± 250 bp, flanked by dotted vertical lines) is significantly higher in the *M. m. casteneus* lineage (red) than in the *M. m. domesticus* lineage (blue; Student test p-value = 4×10^{-4}).
