# Supplementary material for: In vivo binding of PRDM9 reveals interactions with noncanonical genomic sites
Source: Genome Res. 2017 Apr;27(4):580–90. doi: 10.1101/gr.217240.116 (PMC5378176; doi:10.1101/gr.217240.116)
Supplement: Supplemental Material [file supp_gr.217240.116_Supplemental_Fig_S4.pdf]

Supplemental Figure 4

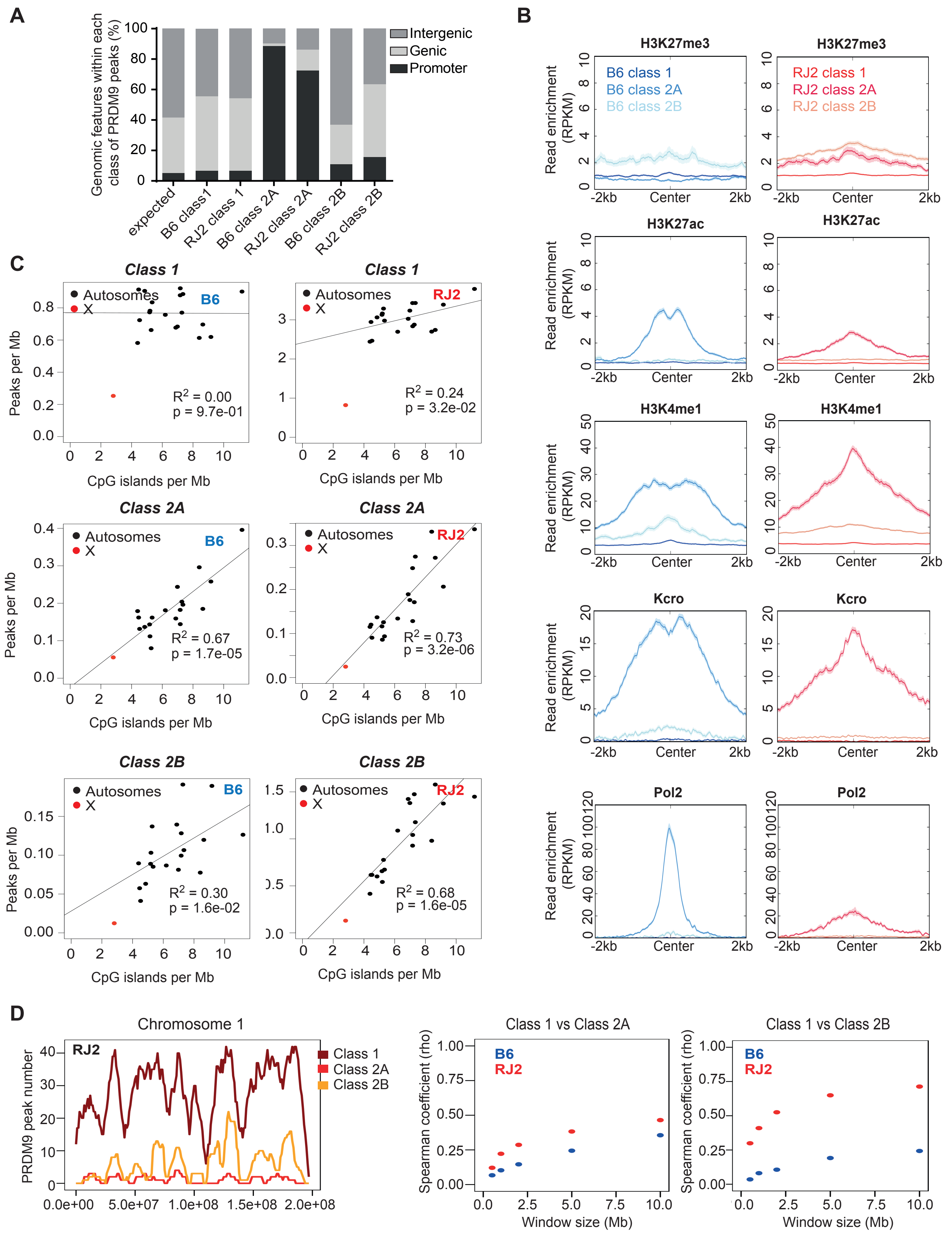

Supplemental Figure S4 The genomic context and the chromatin landscape of PRDM9 class 2 sites differs from canonical class 1 sites. (A) Histogram representing the relative distributions of PRDM9 class 1, 2A and 2B peaks in genic, intergenic and promoter (transcription start sites +/- 1kb) regions compared to the expected random distribution of peaks over the genome. (B) Average read enrichment of various histone posttranslational modifications and Pol2 ChIP-seq centered on PRDM9 class 1, 2A and 2B peaks in B6 (left panels) and RJ2 (right panels) mice. ChIP-seq data for H3K27me3, H3K27ac and H3K4me1 (GSE49624) are from sorted B6 spermatocytes (Hammoud et al. 2014). ChIP-seq data for Kcro (GSE32663) are from B6 germ cells (Tan et al. 2011) and data for Pol2 are for whole adult B6 testes (ENCODE database GSM918704). (C) Peak density per chromosome compared to CpG island density for PRDM9 class 1, 2A and 2B sites of B6 (left panels) and RJ2 (right panels) mice. Each black dot represent an autosome and red dots are Chromosome X. Chromosome X was removed to calculate the Pearson correlation coefficient (R). Regression lines (black) were drawn considering autosomes only. (D) Distribution of $PRDM9^{Cst}$ class 1, 2A and 2B peaks along the Chromosome 1 for RJ2 (left panel, x axis in bp). Peaks were counted in 10Mb window with a 1Mb-step. This distribution is poorly correlated with that of randomized samples (Spearman correlation coefficient lower than 0.38). The correlation (Spearman correlation coefficient, rho) between class 1 and class 2A peaks (middle panel) and class 1 and class 2B peaks (right panel) over the whole genome increases with window size.
