# Supplementary material for: In vivo binding of PRDM9 reveals interactions with noncanonical genomic sites
Source: Genome Res. 2017 Apr;27(4):580–90. doi: 10.1101/gr.217240.116 (PMC5378176; doi:10.1101/gr.217240.116)
Supplement: Supplemental Material [file supp_gr.217240.116_Supplemental_Fig_S5.pdf]

Supplemental Figure 5

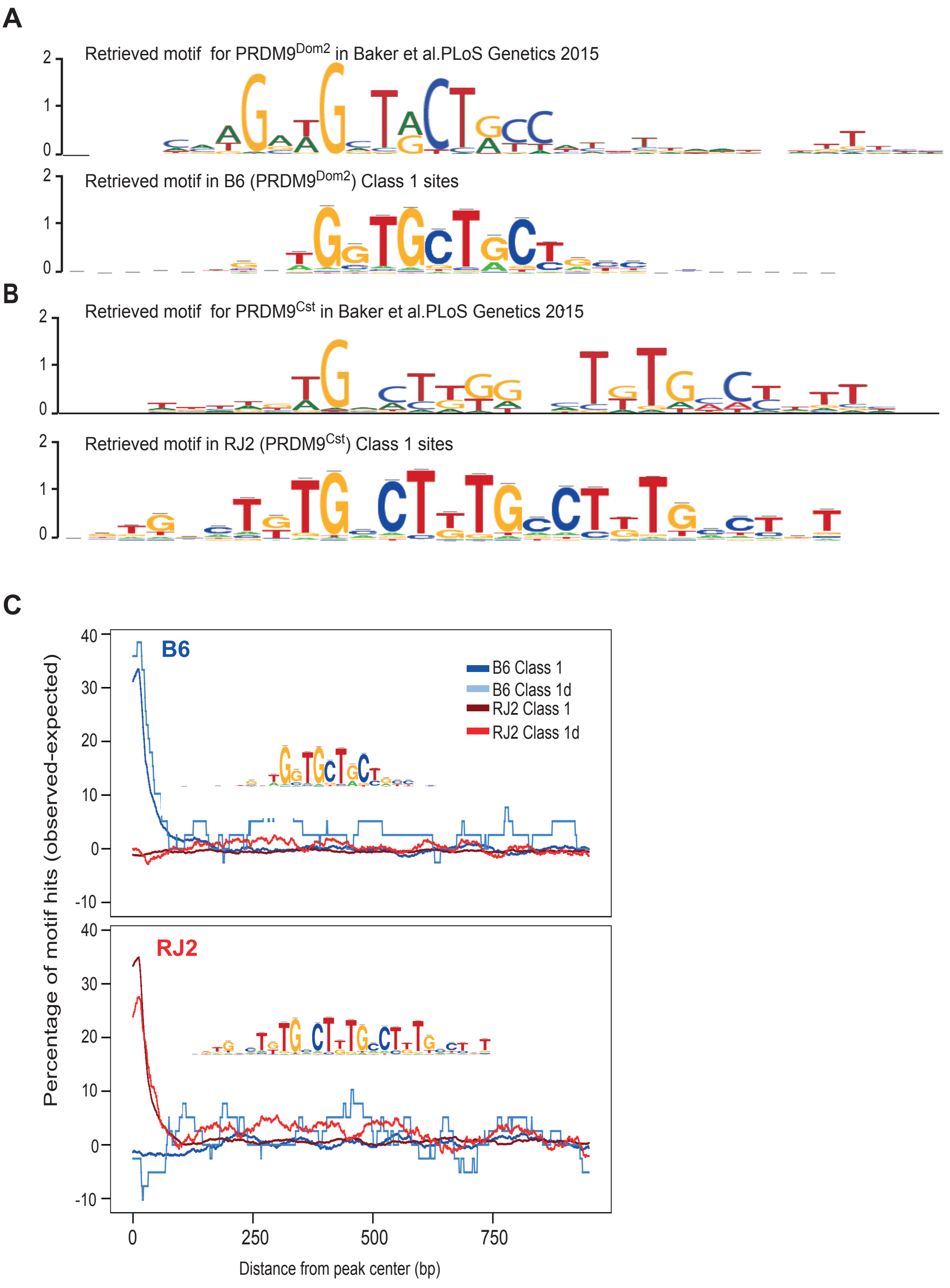

Supplemental Figure S5 Consensus motifs retrieved in B6 (A) and RJ2 (B) PRDM9 class 1 peaks compared to motifs previously found in (Baker et al. 2015). (C) PRDM9 class 1d are enriched in a PRDM9 allele-specific motif. Distribution of hits to PRDM9 *Dom2* and PRDM9 *Cst* motifs (each consensus motif is depicted on each graph) along B6 and RJ2 class 1 (total) and class 1d sites from the center of the PRDM9 sites to 1kb distance. Hits were calculated in a 50bp-sliding window with a 1bp step.
