# Supplementary material for: In vivo binding of PRDM9 reveals interactions with noncanonical genomic sites
Source: Genome Res. 2017 Apr;27(4):580–90. doi: 10.1101/gr.217240.116 (PMC5378176; doi:10.1101/gr.217240.116)
Supplement: Supplemental Material [file supp_gr.217240.116_Supplemental_Fig_S6.pdf]

Supplemental Figure 6

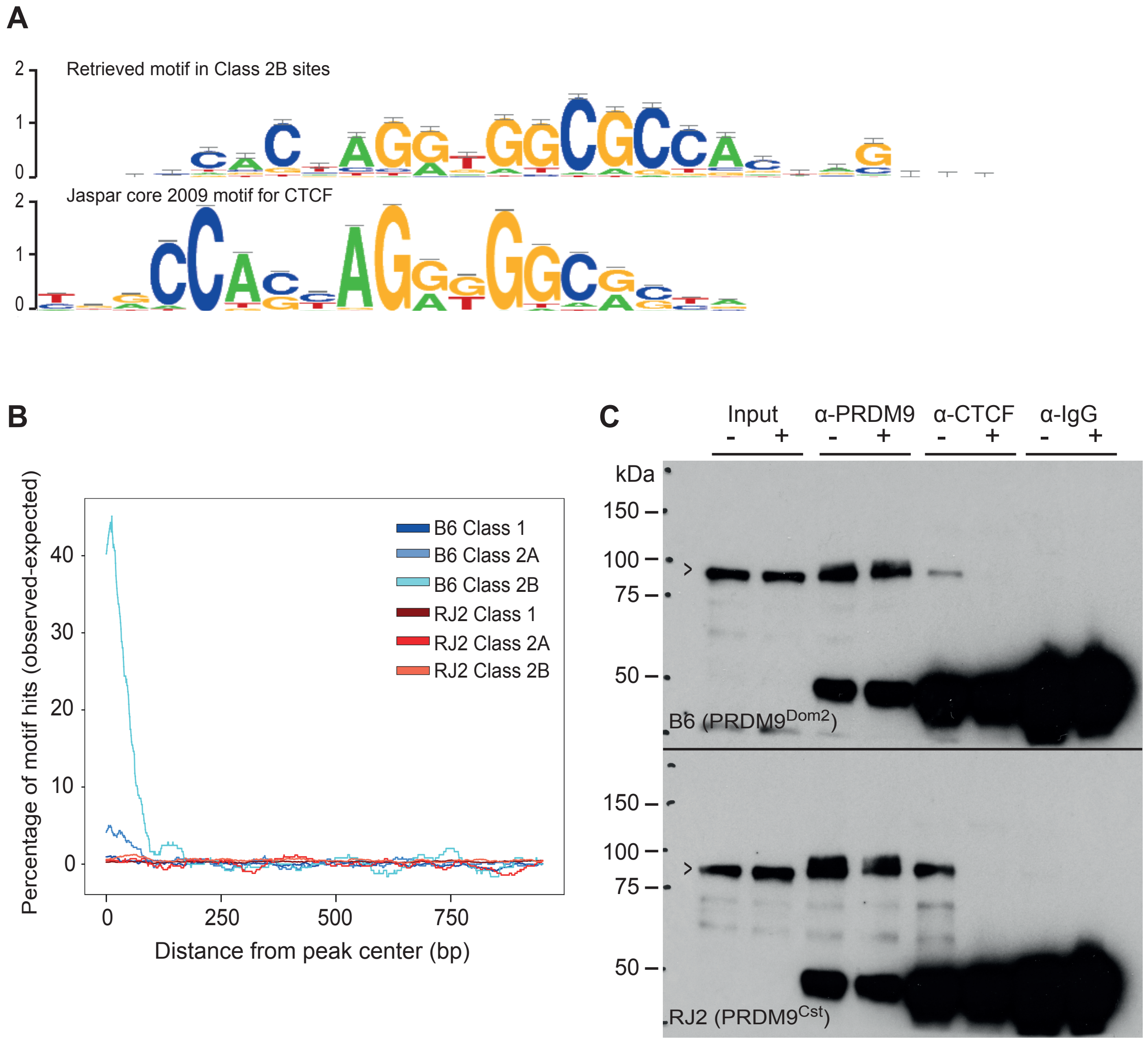

Supplemental Figure S6 PRDM9 interacts indirectly with CTCF. (A) CTCF motif found in B6 class 2B sites compared to Jaspar core (2009) motif retrieved for CTCF in human, which is highly similar to the mouse one. (B) Distribution of CTCF motif hits (using the matrix from Jaspar core 2009) along B6 and RJ2 class 1, 2A and 2B sites from the center of the PRDM9 sites up to 1kb distance. Hits were calculated in a 50bp-sliding window with a 1bp step. (C) Co-Immunoprecipitation assays with juvenile testes extract using anti-PRDM9, anti-CTCF and rabbit serum (anti-IgG) for immunoprecipitation and using anti-PRDM9 for western blot. PRDM9 is indicated by an arrow head. Extracts are treated (+) or not (-) with benzonase before immunoprecipitation. For the samples immunoprecipitated with anti-PRDM9, the equivalent of 30µg of immunoprecipitated extract was loaded and for the samples immunoprecipitated with anti-CTCF or rabbit serum the equivalent of 300µg immunoprecipitated extracts were loaded.
