# Supplementary material for: In vivo binding of PRDM9 reveals interactions with noncanonical genomic sites
Source: Genome Res. 2017 Apr;27(4):580–90. doi: 10.1101/gr.217240.116 (PMC5378176; doi:10.1101/gr.217240.116)
Supplement: Supplemental Material [file supp_gr.217240.116_Supplemental_Fig_S7.pdf]

Supplemental Figure 7

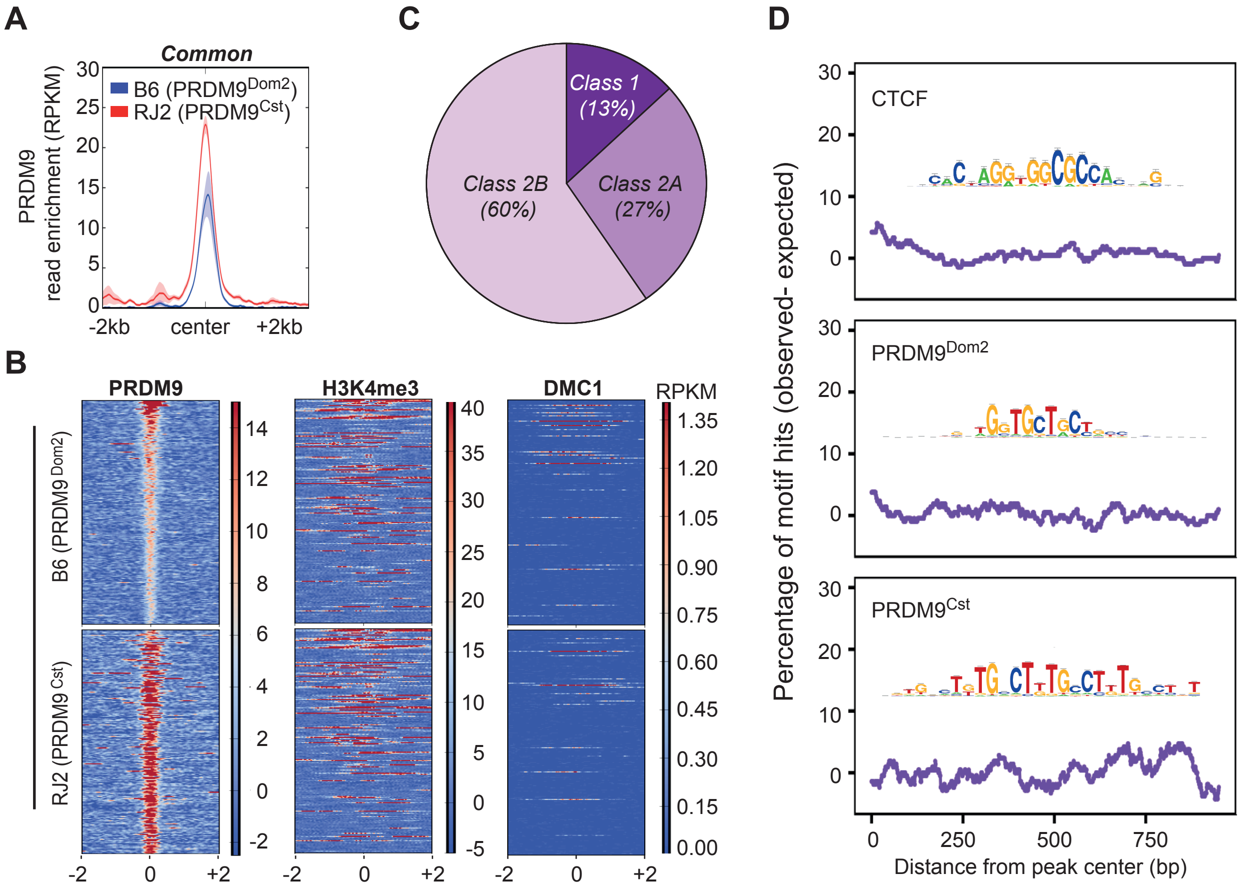

Supplemental Figure S7 PRDM9 sites, shared between B6 and RJ2 strains, are mostly class 2 sites. (A) Average read enrichment (RPKM: reads per kilo base pair per million mapped reads) of PRDM9 in B6 and RJ2 mice centered on sites shared between the two mouse strains (common). (B) Heatmaps of PRDM9, H3K4me3 and DMC1 read distribution centered on sites shared between B6 and RJ2 mice. (C) Pie chart showing the percentage of each class of B6 and RJ2 common peaks. (D) B6 and RJ2 common peaks are not enriched in CTCF or PRDM9 allele-specific motifs. Distribution of hits to CTCF (top), PRDM9Dom2 (middle) and PRDM9Cst (bottom) motifs (each consensus motif is depicted on each graph) along B6 and RJ2 shared sites from the center of the PRDM9 sites up to a distance of 1kb. Hits were calculated in a 50bp-sliding window with a 1bp step.
