# Supplementary material for: In vivo binding of PRDM9 reveals interactions with noncanonical genomic sites
Source: Genome Res. 2017 Apr;27(4):580–90. doi: 10.1101/gr.217240.116 (PMC5378176; doi:10.1101/gr.217240.116)
Supplement: Supplemental Material [file supp_gr.217240.116_Supplemental_Fig_S8.pdf]

Supplemental Figure 8

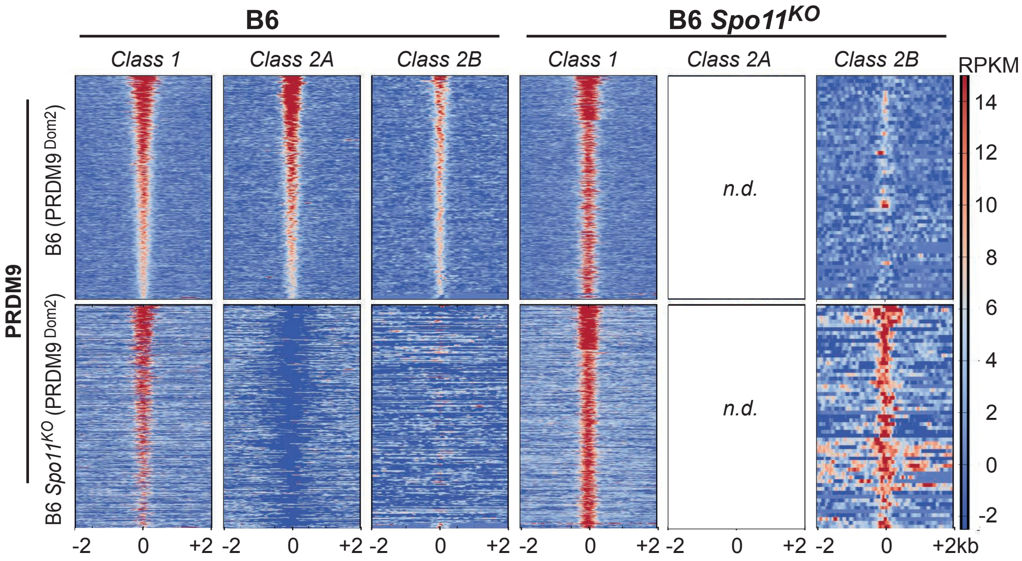

Supplemental Figure S8 In B6 *Spo11*^{KO} mice, class 2A are absent. Heatmaps of PRDM9 read distribution centered on class 1, 2A and 2B PRDM9 peaks in B6 and B6 *Spo11*^{KO} mice, n.d. : not determined (only one class 2A peak was identified in B6 *Spo11*^{KO} mice).
