# Supplementary material for: In vivo binding of PRDM9 reveals interactions with noncanonical genomic sites
Source: Genome Res. 2017 Apr;27(4):580–90. doi: 10.1101/gr.217240.116 (PMC5378176; doi:10.1101/gr.217240.116)
Supplement: Supplemental Material [file supp_gr.217240.116_Supplemental_Fig_S9.pdf]

Supplemental Figure 9

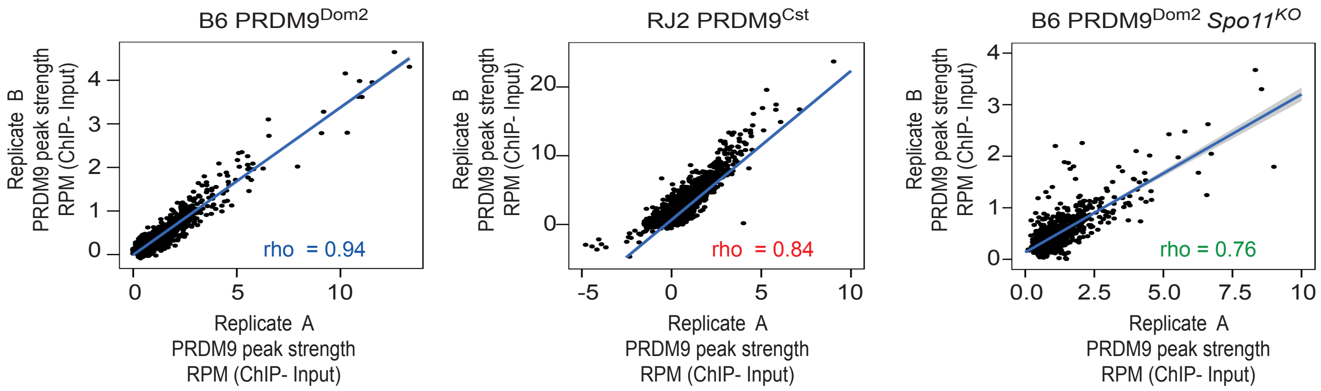

Supplemental Figure S9 PRDM9 ChIP-seq in B6, RJ2 and B6 *Spo11*^{KO} mice are reproducible. Scatter plots of PRDM9 strength (RPM) in replicate A versus replicate B. Left: B6, Middle: RJ2, Right: B6 *Spo11*^{KO}. Rho is the Spearman correlation coefficient.
