# Supplementary material for: In vivo binding of PRDM9 reveals interactions with noncanonical genomic sites
Source: Genome Res. 2017 Apr;27(4):580–90. doi: 10.1101/gr.217240.116 (PMC5378176; doi:10.1101/gr.217240.116)
Supplement: Supplemental Material [file supp_gr.217240.116_Supplemental_Table_S1.pdf]

List of ChIP-seq experiments performed

	Antibody	Experiment	Replicate	Sequencing	Read Number	Accession Number
**B6**	PRDM9	ChIPseq	1	PE 100bp	162Mx2	GSM2465574
**B6**	PRDM9	Input	1	PE 100bp	152Mx2	GSM2465575
**B6**	PRDM9	ChIPseq	2	PE 100bp	168Mx2	GSM2465576
**B6**	PRDM9	Input	2	PE 100bp	158Mx2	GSM2465577
**RJ2**	PRDM9	ChIPseq	1	PE 100bp	94Mx2	GSM2465578
**RJ2**	PRDM9	Input	1	PE 100bp	115Mx2	GSM2465579
**RJ2**	PRDM9	ChIPseq	2	PE 100bp	136Mx2	GSM2465580
**RJ2**	PRDM9	Input	2	PE 100bp	113Mx2	GSM2465581
**B6_PRDM9_KO**	PRDM9	ChIPseq	1	SE 50bp	186M	GSM2465582
**B6_PRDM9_KO**	PRDM9	Input	1	SE 50bp	154M	GSM2465583
**B6_SPO11_KO**	PRDM9	ChIPseq	1	PE 100bp	104Mx2	GSM2465584
**B6_SPO11_KO**	PRDM9	Input	1	PE 100bp	104Mx2	GSM2465585
**B6_SPO11_KO**	PRDM9	ChIPseq	2	PE 100bp	117Mx2	GSM2465586
**B6_SPO11_KO**	PRDM9	Input	2	PE 100bp	97Mx2	GSM2465587
**B6**	H3K4me3	ChIPseq	1	SE 36bp	44Mx2	GSM2465588
**B6**	H3K4me3	Input	1	SE 36bp	46Mx2	GSM2465589
**RJ2**	H3K4me3	ChIPseq	1	SE 36bp	44Mx2	GSM2465590
**RJ2**	H3K4me3	Input	1	SE 36bp	46Mx2	GSM2465591
**B6**	DMC1	SSDS	1	PE 50bp	120Mx2	GSM2465592
**B6**	DMC1	SSDS	2	PE 50bp	142Mx2	GSM2465593
**RJ2**	DMC1	SSDS	1	PE 50bp	122Mx2	GSM2465594
**RJ2**	DMC1	SSDS	2	PE 50bp	126Mx2	GSM2465595
**B6**	H3K36me3	ChIPseq	1	SE 50bp	182M	GSM2465596
**B6**	H3K36me3	Input	1	SE 50bp	153M	GSM2465597
**B6**	H3K36me3	ChIPseq	2	SE 50bp	114M	GSM2465598
**B6**	H3K36me3	Input	2	SE 50bp	173M	GSM2465599
**RJ2**	H3K36me3	ChIPseq	1	SE 50bp	178M	GSM2465600
**RJ2**	H3K36me3	Input	1	SE 50bp	152M	GSM2465601
**RJ2**	H3K36me3	ChIPseq	2	SE 50bp	244M	GSM2465602
**B6_SPO11_KO**	H3K36me3	ChIPseq	1	SE 50bp	154M	GSM2465603
**B6_SPO11_KO**	H3K36me3	Input	1	SE 50bp	148M	GSM2465604
**B6_SPO11_KO**	H3K36me3	ChIPseq	2	SE 50bp	131M	GSM2465605

Supplemental Table S1 Summary of all ChIP-seq experiments performed, indicating genotype, antibodies, sequencing conditions used, number of mapped reads and accession numbers.
