# Supplementary material for: In vivo binding of PRDM9 reveals interactions with noncanonical genomic sites
Source: Genome Res. 2017 Apr;27(4):580–90. doi: 10.1101/gr.217240.116 (PMC5378176; doi:10.1101/gr.217240.116)
Supplement: Supplemental Material [file supp_gr.217240.116_Supplemental_Information.docx]

Supplemental Material

Supplemental Methods

**Antibodies**

Antibodies against PRDM9 were raised by immunizing two rabbits with recombinant full-length PRDM9^Cst^ protein fused to 6x HIS at the N-terminus. PRDM9 polyclonal serum was then affinity-purified by incubation with PRDM9^Dom2^ recombinant protein immobilized on a nitrocellulose membrane. Guinea pig anti-SYCP3 ([Grey et al. 2009](#_ENREF_3)), rabbit anti-SYCP1 (Abcam, 15090) and mouse monoclonal anti-phospho-Histone H2A.X (Ser139) antibody (Upstate-Milipore, 05-636) were used for immunostaining. For DMC1 ChIP, goat anti-DMC1 (Santa Cruz, C-20) was used and for H3K4me3 and H3K36me3 ChIP rabbit anti-H3K4me3 (Abcam, ab8580) and rabbit anti-H3K36me3 (Diagenode, Premium, C15410192) respectively were used. For co-immunoprecipitation of PRDM9 with CTCF we used rabbit anti-CTCF (Cell signaling, D31H2XPR).

**Nuclei spreads and immunostaining**

Spermatocyte nuclei spreads were performed by the dry down technique as described ([Peters et al. 1997](#_ENREF_8)) and immunostaining was performed as described ([Grey et al. 2009](#_ENREF_3)). PRDM9 affinity purified antibody was used at a dilution of 1:100. For staging experiments in Supplemental Figure S1D the criteria were the following: Only SYCP3 positive nuclei were counted, pre-leptotene nuclei had weak SYCP3 signal and no or very weak γH2Ax signal, leptotene nuclei were positive for γH2Ax but negative for SYCP1, early/mid zygotene nuclei had less than nine fully synapsed chromosomes, late zygotene had nine or more chromosomes fully synapsed and pachytene cells had all chromosomes fully synapsed, excepted for the sex chromosomes.

**Histological cryosections and immunostaining**

Testes were fixed for 40min at room temperature in 4% paraformaldehyde, 100mM NaHPO4 pH7.4, 0.1% Triton, then placed in 30% sucrose overnight at 4°C. The testes were then embedded in OCT and cut in 9µm sections. For immunostaining, sections were dried, washed and then incubated for 30min at room temperature in 10% goat serum, 0,05% Tween, 0.1% Triton. Primary antibodies (anti-SYCP3, dilution 1:200 and anti-PRDM9 dilution 1:50) were diluted in blocking buffer and incubated overnight at room temperature. Secondary antibodies were also diluted in blocking buffer and incubated for 1 hour at room temperature.

**PRDM9 extraction and western blotting**

Nuclear extracts were obtained by Dignam extraction. Briefly, testes were decapsulated and homogenized in 10MM HEPES-Na pH7.4, 320mM sucrose, 2mM PMSF, EDTA-free 1x complete protease inhibitor (Roche, 11873580001), 0.07% β-mercaptoethanol, and centrifuged at 1000g for 10min to remove the cytoplasmic fraction. The pellet was resuspended in a low-salt buffer (20mM Tris-HCl pH7.3 12.5% glycerol 1.5mM MgCl2, 0.2mM EDTA, 20mM KCl, 2mM PMSF, EDTA-free 1x complete protease inhibitor, 0.07% β-mercaptoethanol), and then the same amount of high-salt buffer (20mM Tris-HCl pH7.3 12.5% glycerol 1.5mM MgCl2, 0.2mM EDTA, 1.2M KCl, 2mM PMSF, EDTA-free 1x complete protease inhibitor, 0.07% β-mercaptoethanol) was added dropwise before incubating the extract for 30min at 4°C. The sample was then centrifuged for 30min at 18,000g at 4°C and the supernatant was retrieved. Western blotting was performed as follows: 25µg of extract was loaded on a 7.5% TBX-acrylamide gels (Biorad, 4561023S) and then blotted on nitrocellulose membranes, blocked and incubated o/n at 4°C with affinity purified anti-PRDM9 antibody (1:1000), then with donkey anti-rabbit-HRP conjugated antibody (Jackson, 711-035-152) and visualized with pico ECL substrate (Pearce, Thermo Fisher, 32196).

**Immunoprecipitation of *in vitro* translated PRDM9**

Full length *Prdm9^Dom2^* and *Prdm9^Cst^* were cloned into pET15 (Novagen, EMD Millipore, 69661) to produce His (6x)-tagged fusion proteins. *In vitro* translation was performed as in ([Kumar et al. 2010](#_ENREF_6)). For immunoprecipitation, 4μL of each *in vitro* translated protein was incubated overnight at 4°C in 250μL of HNTG buffer (20mM Hepes, 150mM NaCl, 1% Triton, 10% Glycerol, 1mM MgCl2, 1mM EGTA, protease inhibitor cocktail, Roche) with anti-PRDM9 antibody (1:50 dilution). Next, the samples were incubated for 2 hours with 10µL of protein A dynabeads (Thermo Fisher, 10002D). After five washes with HNTG buffer, samples were loaded on a SDS-PAGE gel which was then fixed, dried and immediately exposed for 72 hours.

**Co-Immunoprecipitation of PRDM9 and CTCF**

Dignam nuclear extracts from juvenile (12dpp or 13dpp) testes were incubated overnight with 0.6µg of antibody (Anti-PRDM9, anti-CTCF or rabbit Serum IgG). Protein-antibody complexes were pulled down with protein G Dynabeads (Thermo Fisher, 10003D). Immunoprecipitated extracts were separated by electrophoresis, blotted and visualized (anti-PRDM9) as described above.

**Chromatin Immunoprecipitation of PRDM9 and H3K36me3**

In order to perform the detection of PRDM9 binding to chromatin in optimal conditions, we purified chromatin from testis of young mice, where the relative abundance of early stages of meiotic prophase is elevated. Indeed, a first wave of entry of spermatogonia into meiosis initiates at 8 days postpartum (8dpp) and spermatocytes progress into meiotic prophase and simultaneously reach the leptotene, zygotene and pachytene stages at approximately 11, 13 and 15dpp such that the proportion of cells at leptotene/zygotene is 55, 41 and 26% at these three ages respectively ([Goetz et al. 1984](#_ENREF_2)).

Testes from ten 13dpp mice were de-capsulated and fixed for 10 min in 1% formaldehyde (4 juvenile mice were used for H3K36me3). After quenching the reaction, tissue was homogenized and a cell suspension was prepared by filtering through a 40µm cell strainer and washing twice in 60mM KCl, 0.5% Triton, 15mM HEPES pH8, 4mM MgCl2, 15mM NaCl, 0.5mM DTT, 1x complete protease inhibitor cocktail EDTA-free (Roche, 11873580001). Cells were lysed in 50mM HEPES pH8, 1% SDS, 1mM EDTA, 1mM PMSF, 1x complete protease inhibitor cocktail EDTA-free for 30 minutes on ice. Sonication was performed to shear chromatin to fragments of about 350 base pairs. Sonicated chromatin was then concentrated eight-fold on 10,000-Vivaspin 4 columns (Sartorius, GH Healthcare, 28932296) and then diluted tenfold in IP buffer: 1% Triton, 50mM HEPES, 150mM NaCl, 1mM EDTA, 1mM PMSF, 1x complete protease inhibitor cocktail EDTA-free (Roche) and pre-cleared with 35µL of protein A-or G-Dynabeads (Thermo Fisher, 10002/3D) for 4h. Chromatin was then incubated overnight at 4°C with 20µg of anti-PRDM9 and with 10µg of anti-H3K36me3. Samples were then incubated for several hours with 100µL of Dynabeads and washed once in each of the following buffers: Wash I; 1% Triton, 150mM NaCl, 20mM HEPES, 0,1% SDS, 1mM EDTA, 1mM PMSF, Wash II; 1% Triton, 500mM NaCl, 20mM HEPES, 0,1% SDS, 1mM EDTA, 1mM PMSF, Wash III; 125mM LiCl, 1% Deoxycholic acid, 1% NP40, 10mM HEPES, 1mM EDTA. Elution was performed for 40 minutes at 65°C in 1% SDS and 100mM NaHCO3 and crosslinking was reversed overnight at 65°C. After incubation with proteinase K, DNA was purified with MiniElute columns (Qiagen, 28004).

**Library construction and sequencing**

Standard library construction was performed with the Illumina TruSeq protocol (Illumina, IP-202-9001DOC). For DMC1 ChIP, an additional step of kinetic enrichment was performed, as described in ([Khil et al. 2012](#_ENREF_5)). Sequencing was performed on a HiSeq2000 (Illumina) or on a Genome Analyzer II (for H3K4me3 ChIP-sequencing experiment)

**Computational Data Analysis**

For PRDM9, H3K4me3 and H3K36me3 ChIP-seq data processing, reads were mapped to the UCSC mouse genome assembly, build mm9, using Bowtie2 (parameters for PE reads: -k2 -no-misc -no-disc ; parameters for SE reads : -k2). The analysis of DMC1 ChIP-seq was performed as described ([Khil et al. 2012](#_ENREF_5)), using their modified BWA algorithm and their customed script, specifically developed to align and recover ssDNA fragments. A filtering step was performed on aligned reads for all ChIP experiments keeping non-duplicated and high-quality uniquely mapped reads with no more than one mismatch per read. To identify enriched regions from biologically replicated samples in PRDM9 and DMC1 ChIP-seq, we used the IDR (Irreproducible Discovery Rate) methodology that has been developed for ChIP-seq analysis and extensively used by the ENCODE and modENCODE projects ([Landt et al. 2012](#_ENREF_7)). We followed the framework developed by Qunhua Li and Peter Bickel's group (https://sites.google.com/site/anshulkundaje/projects/idr). Briefly, this method allows testing the reproducibility within and between replicates by using the IDR statistics. Following their pipeline, peak calling was performed using MACS version 2.0.10 with relaxed conditions (PRDM9: --pvalue1e-2 --mfold 2-50 --bw300 ; DMC1 : --pvalue1e-1 --bw1000 --nomodel --shift400 ) on each of the two replicates, the pooled dataset, and on pseudo replicates that were artificially generated by randomly sampling half the reads twice for each replicate and the pooled dataset. Then IDR analysis was performed and reproducibility was checked. Final peak sets were built by picking up the top N peaks from pooled dataset (ranked by increasing p values), with N defined as the maximum between the peak number below an IDR threshold of 0.01 for pooled dataset and the peak number below an IDR threshold of 0.05 for true replicates, as recommended by the authors. Peak calling for the PRDM9^KO^ ChIP-seq experiment was performed using MACS version 2.0.10 with a relaxed p-value (compared to the highest pvalue of 1e-11 obtained for B6 and RJ2, after IDR analysis) to get the most comprehensive control dataset possible (parameters --pvalue1e-3 --mfold 2-50 --bw300). We then tested the correlation between PRDM9 peak strength of the two replicates for specific peaks of B6, B6 SPO11^KO^ and RJ2 (Supplemental Fig. S9).

H3K4me3 ChIP-seq reads were mapped to the UCSC mouse genome assembly, build mm9, using Bowtie (parameters: -q --best -v 2 -I 100 -X 1000). Peak calling was then performed on three different sets of paired reads (all successfully aligned reads, non-duplicated and successfully aligned reads, uniquely aligned reads without any mismatch) using MACS1.3.7 (parameters: --pvalue=1e-4 --mfold=8 --space=50). The most robust peaks were then defined as the intersect peak calling results from these three sets of paired reads.

All read distribution and peak strength presented in this work were calculated for each experiment on pooled replicates in 1-bp windows by normalizing the signal by the total library size then subtracting input signal.

**Determination of overlapping peaks and classes**

PRDM9 peaks with unspecific signal were first removed from each B6, B6 SPO11^KO^ and RJ2 previously defined peak-set by taking off those having a 1bp-overlap of the interval with peaks called from the B6 PRDM9^KO^ ChIP-seq. To test for the presence of H3K4me3 marks and/or of a double strand breaks near PRDM9 binding sites, each previously defined PRDM9 peak-set was overlapped with H3K4me3 and DMC1 peak-sets. PRDM9 peaks were thus overlapped with strain-specific and common H3K4me3 sites, using a 10%-overlap threshold (10% of the PRDM9 peak size has to overlap to get a positive overlap). In addition to our datasets, we also used the H3K4me3 datasets that were previously published as control ([Baker et al. 2014](#_ENREF_1)) for B6 and for B6-Prdm9^CAST-KI^/Kpgn, a strain with a B6 genetic background expressing PRDM9^Cst^ similar to RJ2. Overlaps with DSB hotspot positions were restricted to the central 400bp of DMC1 peaks. Classes of PRDM9 binding were then defined as shown in Figure 1D and 5F.

**Analysis of binding motifs**

For each PRDM9 binding class, we looked for specific enriched motifs within 1kb around peak centers using the peak-motifs tool from RSAT (Regulatory Sequence Analysis Tool), with oligo-analysis, position-analysis and local-word-analysis for k-mers of length 6 and 7 turned on. Motifs identified were compared to cisBP mouse, Hocomoco (Mouse Tfs), and JASPAR core non-redundant vertebrate databases. In class 1 sites, we identified specific motifs similar to those previously described ([Smagulova et al. 2011](#_ENREF_9); [Baker et al. 2014](#_ENREF_1)) and constructed the consensus motif using the matrix-clustering tool of RSAT. We then used all the matrices obtained from the peak-motif analysis to scan peaks from each class in each strain to determine motif distribution along peaks. Using a 50bp-sliding windows with a 1bp-step, we counted the number of peaks having at least one match in this window. We choose a p-value lower than 10^-5^, and we normalized by the number of hits found on the corresponding control file (i.e. random distribution of the peak-set in the genome) and by the total number of peaks in the given dataset. Different values for sliding-window, step-size and p-value were tested and led to similar results (data not shown).

**GC-biased gene conversion signature analysis**

Genomic sequences from *M. m. castaneus* (CAST/EiJ) and from *Mus spretus* (SPRET/EiJ) ([Keane et al. 2011](#_ENREF_4)) mapped on the *M. m. domesticus* reference genome assembly were retrieved from <ftp://ftp-mouse.sanger.ac.uk/>. Substitutions having occurred along the *M. m. castaneus* lineage or along the *M. m. domesticus* lineage were identified by comparison with the *M. spretus* outgroup. The equilibrium GC-content (GC*) in a given lineage was computed as:

${GC}^{*}=\frac{u}{u+v}$

where *u* is the AT→GC substitution rate in that lineage (number of AT→GC substitutions per ancestral AT site) and *v* is the GC→AT substitution rate that lineage (number of GC→AT substitutions per ancestral GC site). Student t tests were performed on GC* within peak center ± 250 bp.

**Analysis of peak distribution along chromosomes**

For each PRDM9 binding site class, we determined the peak number within increasing window sizes (0.5Mb, 1Mb, 2Mb, 5Mb, 10Mb) and calculated the correlation between distributions by using the Spearman correlation rank. Distributions along chromosomes were plotted for a window of 10Mb with a 1Mb-step.

Supplemental references

Baker CL, Walker M, Kajita S, Petkov PM, Paigen K. 2014. PRDM9 binding organizes hotspot nucleosomes and limits Holliday junction migration. *Genome Res* **24**: 724-732.

Goetz P, Chandley AC, Speed RM. 1984. Morphological and temporal sequence of meiotic prophase development at puberty in the male mouse. *Journal of Cell Science* **65**: 249-263.

Grey C, Baudat F, de Massy B. 2009. Genome-Wide Control of the Distribution of Meiotic Recombination. *PLoS Biol* **7**: e35.

Keane TM, Goodstadt L, Danecek P, White MA, Wong K, Yalcin B, Heger A, Agam A, Slater G, Goodson M et al. 2011. Mouse genomic variation and its effect on phenotypes and gene regulation. *Nature* **477**: 289-294.

Khil PP, Smagulova F, Brick KM, Camerini-Otero RD, Petukhova GV. 2012. Sensitive mapping of recombination hotspots using sequencing-based detection of ssDNA. *Genome Res* **22**: 957-965.

Kumar R, Bourbon HM, de Massy B. 2010. Functional conservation of Mei4 for meiotic DNA double-strand break formation from yeasts to mice. *Genes Dev* **24**: 1266-1280.

Landt SG, Marinov GK, Kundaje A, Kheradpour P, Pauli F, Batzoglou S, Bernstein BE, Bickel P, Brown JB, Cayting P et al. 2012. ChIP-seq guidelines and practices of the ENCODE and modENCODE consortia. *Genome Res* **22**: 1813-1831.

Peters AH, Plug AW, van Vugt MJ, de Boer P. 1997. A drying-down technique for the spreading of mammalian meiocytes from the male and female germline. *Chromosome Res* **5**: 66-68.

Smagulova F, Gregoretti IV, Brick K, Khil P, Camerini-Otero RD, Petukhova GV. 2011. Genome-wide analysis reveals novel molecular features of mouse recombination hotspots. *Nature* **472**: 375-378.
